# Interleukin-39 aggravates sepsis by inducing self-sustaining neutrophil activation in the lungs via the CXCL1-CXCR2 pathway

**DOI:** 10.7150/ijbs.124607

**Published:** 2026-07-22

**Authors:** Jinming Zhang, Jingting Liu, Yihui Wang, Yifan Xu, Robim M Rodrigues, Zihan Zhou, Jie Lu, Ziqiang Li, Xiaolin Wang, Jingjiao Li, Yong He, Fouad Lafdil, Ruidong Mo, Enqiang Mao, Qing Xie, Xiaogang Xiang

**Affiliations:** 1 Department of Infectious Diseases, Translational Laboratory of Liver Diseases, Ruijin Hospital, Shanghai Jiao Tong University School of Medicine, Shanghai, 200025, China.; 2 Department of Emergency, Ruijin Hospital, Shanghai Jiao Tong University School of Medicine, Shanghai, China.; 3 Department of In Vitro Toxicology and Dermato-Cosmetology (IVTD), Faculty of Medicine and Pharmacy, Vrije Universiteit Brussel (VUB), Laarbeeklaan 103, B1090, Brussels, Belgium.; 4 Shanghai Institute of Materia Medica (SIMM), Chinese Academy of Sciences, Shanghai, 201203, China.; 5 Université Paris-Est Créteil (UPEC), Créteil, France.; 6 INSERM, U938, Centre de Recherche Saint-Antoine (CRSA), Paris, France.; 7 Sorbonne University, Paris, France.; 8 Institut Universitaire de France (IUF), Paris, France.

**Keywords:** IL-39, sepsis, systemic inflammation, neutrophil, CXCR2

## Abstract

**Background:**

Sepsis, a life-threatening condition, involves dysregulated host responses to infection that frequently lead to multi-organ failure. Despite advancements in supportive care, current therapies for this disease remain limited, necessitating novel treatment approaches. Interleukin-39 (IL-39), a recently identified member of the IL-12 family, has emerged as a key mediator of inflammatory diseases; however, its functional role in the pathogenesis of sepsis remains largely unknown.

**Methods:**

Serum levels of IL-39 were analyzed in septic patients and healthy individuals. A cecal ligation and puncture (CLP)-induced sepsis murine model was employed. Recombinant human IL-39 (rhIL-39) or an IL-39-neutralizing antibody was administered to CLP mice to investigate both the causative role of IL-39 as well as the anti-IL-39 therapeutic potential. The survival rates, the severity of pulmonary injuries, the levels of pro-inflammatory cytokines, and the infiltration of neutrophils in septic mice were assessed. Bulk RNA sequencing was performed to explore the mechanisms underlying IL-39 promotion of sepsis.

**Results:**

Septic patients had significantly elevated serum levels of IL-39 compared to healthy individuals. The levels of IL-39 were strongly correlated with clinical severity and 90-day mortality in the septic patients. In CLP-induced septic mice, rhIL-39 administration enhanced pulmonary damage, increased systemic inflammation and worsened overall survival. Conversely, IL-39 neutralizing antibodies reversed these effects and improved the survival outcome. Mechanistically, RNA sequencing analysis revealed IL-39 treatment markedly increased several chemokines and pro-inflammatory cytokines, and enriched "cytokine-cytokine receptor interaction" pathway. Finally, rhIL-39 enhanced neutrophil recruitment to lung tissues, a process that was proven to be linked to the CXCL1-CXCR2 pathway activation by *in vitro* analyses.

**Conclusions:**

This study demonstrates that IL-39 is a critical exacerbating factor in sepsis progression, driving neutrophil-mediated tissue injury via CXCL1-CXCR2 signaling. These findings identify IL-39 as both a prognostic biomarker and a promising potential therapeutic target for sepsis management.

## Introduction

Systemic inflammation is prevalent across a variety of diseases and is the hallmark of numerous inflammatory conditions such as Corona Virus Disease 2019 (COVID-19) and acute pancreatitis 1-3. Sepsis is a life-threatening medical condition in which systemic inflammation, triggered by various kinds of pathogens, leads to damage of multiple organs 4, 5. During the hyperinflammatory phase of sepsis, both the innate and adaptive immune systems are activated to eliminate the causative pathogens. However, this initial overactive immune response is often followed by a period of immunosuppression and organ dysfunction, which could result in multiple organ failure (MOF), secondary infections, and ultimately death 6. Despite advancements in supportive care, including early detection and rapid intervention, which have led to a reduction of sepsis mortality over the past decades, the overall 90-day mortality rate remains approximately 35.5% 7. This makes sepsis the leading cause of death among patients in intensive care units (ICU) worldwide 8-12. Although recent advancements in drug development and organ support therapies have been made in recent years, sepsis still remains the leading cause of death among critically ill patients 13. In the early stages of sepsis, systemic inflammatory response syndrome (SIRS) often occurs, which is the major contributor to early mortality 8. Inflammatory cytokines play crucial roles in this systemic inflammatory response, leading to immune-mediated tissue and organ damages 14. Consequently, therapies targeting these inflammatory cytokine-mediated processes have been a central research focus in the treatment of sepsis 15.

Recently, the interleukin (IL)-12 family has attracted substantial attention because of its pivotal roles in inflammatory diseases. The IL-12 family consists of IL-12, IL-23, IL-27, IL-35, and IL-39, all of which are characterized by their composition of two subunits 16, 17. IL-39, the most recently identified member of the IL-12 family, is composed of the IL-23 subunit p19 (IL-23p19, also known as IL-23A) and epstein-barr virus-induced gene 3 (EBi3) subunit. In 2016, Wang *et al.* first reported that IL-39 could aggravate the severity of inflammation in a mouse model of systemic lupus erythematosus (SLE) 18. Moreover, Lv *et al.* found that IL-39 exerts pro-inflammatory effects in mice with graft-versus-host disease (GVHD) 19. Moreover, Sari *et al.* observed that the levels of IL-39 were upregulated in parallel with those of IL-1β and periostin in patients with periodontal disease, indicating that IL-39 might be involved in periodontal inflammation 20. Yet, although an increasing number of studies suggest that IL-39 might play a significant role in pro-inflammatory responses, the precise pathophysiological role of IL-39 in sepsis remains ambiguous.

The present study reveals a pronounced elevation of IL-39 in septic patients, with IL-39 concentrations correlating with levels of inflammatory cytokines, as well as clinical severity scores. In a cecal ligation and puncture (CLP)-induced sepsis model, IL-39 was found to exacerbate systemic inflammation by promoting excessive cytokine release from neutrophils, thereby increasing the mortality risk in septic mice. Notably, inhibition of IL-39 improved survival outcomes in septic mice, highlighting its potential therapeutic value.

## Material and Methods

### Patients and sample preparation

Serum samples from septic patients were obtained from Department of Emergency, Shanghai Ruijin Hospital, affiliated to Shanghai Jiao Tong University School of Medicine. The experiment protocol was approved by Human Ethics Committee of Ruijin Hospital (Ethics committee reference number: 2020-353), and written informed consents were obtained from the subjects. The inclusion criteria were as follows: (i) age ≥ 18 years; (ii) diagnosis of sepsis defined as suspected infection plus organ dysfunction (total SOFA score ≥ 2); (iii) enrollment within 72 hours after the diagnosis of sepsis; (iv) non-pregnant throughout the study period 21.

### Mice

Male C57BL/6J mice (6 to 8 weeks old) were purchased from Beijing Vital River Laboratory Animal Technology Co. Ltd. All the experimental animals were housed in cages (with a maximum of five mice per cage) and maintained in a temperature (24±1°C) and light controlled facility (12 h:12 h light-dark cycle). Animals were randomly assigned to groups and outcome assessors were blinded to treatment.

### CLP model

Polymicrobial sepsis in mice was induced by cecal ligation and puncture (CLP) 22. Briefly, mice were anesthetized by intraperitoneal injection of 1% pentobarbital. The cecum of the anesthetized mice was exposed after a midline laparotomy. The distal portion of exposed cecum (1cm) was ligated with 3-0 silk suture and two punctures were made using a 20G needle for one pass. Subsequently, the cecum was replaced into the abdomen cavity, and the incision was sutured with 4-0 suture. Sham operated mice underwent the same procedures except for cecal puncture. Organ injury scores were calculated and analyzed according to our previously described methods 23.

Two hours after CLP surgery, mice were intraperitoneally administered with either recombinant human IL-39 (rhIL-39; 9990-IL-050, R&D Systems, USA) at 200 μg/kg, dissolved in PBS, or an anti-IL-39 antibody (DETAIBIO, China) at 5 or 8 mg/kg, which has been previously validated 19. To further determine whether CXCR2 signaling contributed to the biological effects of IL-39, additional CLP mice were treated with IL-39 in the presence or absence of the CXCR2 inhibitor SB 265610 (0.5 mg/kg; Selleck Chemicals, Houston, TX, USA) two hours after CLP surgery. SC144 (a small-molecule inhibitor of IL-39-associated receptor IL-6ST/GP130, S7124, Selleck, USA) was administered intraperitoneally at 0 h and 6 h post-CLP induction at a dose of 10 mg/kg. The mice were monitored for survival up to 5 days.

### Real-time quantitative polymerase chain reaction (RT-qPCR)

Total RNA from the tissues was isolated using the Tissue RNA Purification Kit (EZB-RN001-plus, EZBioscience, USA), according to the manufacturer's instructions. cDNA was synthesized from equal amounts of total RNA using Color Reverse Transcription Kit (A0010CGQ-L, EZBioscience, USA). Quantitative real-time PCR was performed using 2×Color SYBR Green qPCR Master Mix (A0012-R2-L, EZBioscience, USA) along with the primers described in **Table S10-S11**. Each reaction was run in triplicate. Gene expression levels were normalized to the housekeeping genes GAPDH and β-actin, and relative mRNA expression was calculated using the 2-ΔΔCt method.

### RNA sequencing

Total RNA from the lung was isolated using the Tissue RNA Purification Kit (EZB-RN001-plus, EZBioscience, USA), according to the manufacturer's instructions. RNA integrity was verified by agarose gel electrophoresis, while quantification and purity assessments were performed via NanoDrop spectrophotometry (Thermo Scientific), with A260/A280 ratios consistently monitored. Only RNA samples meeting stringent quality thresholds (intact bands, A260/A280 ≈ 1.8-2.2) were selected for downstream processing. Subsequent cDNA library construction and RNA sequencing were carried out by Biotree Biotechnology Co., Ltd (Shanghai) using the Illumina Novaseq™ 6000 platform 24, ensuring high-throughput paired-end sequencing with rigorous quality control.

### Western blotting analysis

Mouse spleen tissue was homogenized in lysis buffer to obtain total protein. The concentration of protein was determined by mean of bicinchoninic acid (BCA) protein assay (Thermo Scientific^TM^, USA). Twenty micrograms of total protein were loaded onto a pre-cast SDS-PAGE gel (M00657, GenScript, USA) and then transferred onto nitrocellulose membranes. Immunoblotting was performed using the following antibodies: α-tubulin Rabbit mAb (HRP-66031, Proteintech, USA), GAPDH Rabbit mAb (5174S, Cell Signaling Technology, USA), IL-23A mouse Antibody (107985-T32, Sino Biological, China), EBi3 Rabbit mAb (A19613, Abclonal, China), CXCR2 Rabbit pAb (A3301, Abclonal, China), β-actin Rabbit mAb (4970S, Cell Signaling Technology, USA), STAT1 Rabbit mAb (14994S, Cell Signaling Technology, USA), STAT3 Rabbit mAb (12640S, Cell Signaling Technology, USA), Phospho-STAT1 (Tyr701) Rabbit mAb (9167S, Cell Signaling Technology, USA), Phospho-STAT3 (Tyr705) Rabbit mAb (9145S, Cell Signaling Technology, USA).

For detection of phosphorylated and total STAT proteins, the membranes were first probed with antibodies against phospho-STAT1 (Tyr701) or phospho-STAT3 (Tyr705). After imaging, the membranes were stripped using a commercial stripping buffer (Epizyme, PS107S) according to the manufacturer's instructions and then reprobed with antibodies against total STAT1 or STAT3.

### Hematoxylin & eosin (H&E) staining

Hematoxylin and eosin (H&E) staining was performed in accordance with established histological protocols 25, 26: Lung tissues were fixed in 10% neutral-buffered formalin, paraffin-embedded, and sectioned at 4-μm thickness. Following deparaffinization and rehydration procedures, sections were stained with hematoxylin to visualize nuclei and counterstained with eosin for the visualization of cytoplasmic and extracellular matrix. Subsequently, the slides were dehydrated, cleared, and coverslipped. Histopathological evaluation was carried out under light microscopy, and lung injury was evaluated based on established criteria, such as alveolar damage and inflammatory cell infiltration 23. Representative images were shown, and the regions of injury were quantified in 5 randomly selected microscopic fields (×100) per sample using Image J software (version 1.47v, NIH, USA) 25.

### Terminal deoxynucleotidyl transferase dUTP nick end labeling (TUNEL) staining

Terminal deoxynucleotidyl transferase dUTP nick end labeling (TUNEL) assay was conducted using standardized protocols for histochemical detection of DNA fragmentation 27. Paraffin-embedded lung tissue sections (4 μm) were deparaffinized, rehydrated, and processed for TUNEL staining using a commercial kit (Aifang bio, AFIHC030, China), following the manufacturer's instructions. Sections were treated with proteinase K, incubated with the TUNEL reaction mixture, and counterstained with DAPI to visualize nuclei. Apoptotic cells were identified by fluorescence microscopy, and the proportion of TUNEL-positive cells was calculated in relation to the total number of DAPI-stained nuclei.

### Immunohistochemistry staining

Immunohistochemistry (IHC) staining was performed on pulmonary tissues fixed with 10% neutral buffered formalin, followed by paraffin embedding and sectioning at 4-μm. After xylene deparaffinization and ethanol gradient rehydration, antigen retrieval was achieved using controlled thermal exposure. Endogenous peroxidase was quenched with 3% H₂O₂, followed by incubation with primary antibodies and HRP-conjugated secondaries 23. Immunoreactivity was visualized using DAB chromogen, nuclei were contrasted with hematoxylin, and sections were analyzed by bright-field microscopy.

### Immunofluorescence staining

Immunofluorescence (IF) staining was performed on lung tissues collected from CLP mice 14 h after surgery. Tissues were fixed in 10% neutral-buffered formalin, paraffin-embedded, and sectioned at 4 μm. After deparaffinization in xylene and rehydration through a graded ethanol series, antigen retrieval was performed using heat-induced epitope retrieval. Sections were permeabilized when required and blocked to minimize non-specific binding. The sections were then incubated with primary antibodies against IL-23p19 (anti-IL-23p19, HY-P990217, MedChemExpress, USA) and EBI3 (anti-EBI3, MABF848, Merck Millipore, USA) to assess their spatial co-localization in septic lung tissues. After washing, sections were incubated with species-appropriate fluorophore-conjugated secondary antibodies in the dark. Nuclei were counterstained with DAPI, and slides were mounted using an antifade mounting medium. Fluorescence images were acquired using a fluorescence microscope under identical acquisition settings across all groups to allow direct comparison.

### Enzyme-linked immunosorbent assay (ELISA)

Whole blood from patients and mice was collected, and the serum was separated by centrifugation, and stored at -80 °C. Serum IL-39 was quantified by enzyme-linked immunosorbent assay (ELISA) using the Human IL-39 kit (MBS8808094, MyBioSource, USA) and the Mouse IL-39 kit (DRE-M0517c, RapidBio, USA), respectively 19. Tissue IL-6, IL-1β, and TNF-α levels were measured using the Mouse IL-6 One-Step ELISA kit (EK206EGA, MultiSciences, China), the Mouse IL-1β One-Step ELISA kit (EK201BEG, MultiSciences, China), and the Mouse TNF-α One-Step ELISA kit (EK282EG, MultiSciences, China), respectively. All samples were measured in duplicate, and the results were expressed as pg/mL.

### Cell culture

HL60 cells were maintained in RPMI 1640 medium (Servicebio, G4538-500mL, China) containing 10% FBS, and they were induced to differentiate using 1.25% dimethyl sulfoxide (DMSO, Thermo Fisher Scientific, USA). RAW264.7 cells were cultured in high-glucose DMEM (Servicebio, G4511-500mL, China) supplemented with 10% fetal bovine serum (FBS, Meilun Bio, China). It is reported that rhIL-39 exhibits biological activity at concentrations of 20-50 ng/mL 19. Accordingly, 50 ng/mL was selected, and 100 ng/mL was included to evaluate near-maximal responses while monitoring cell viability. The differentiated HL60 (dHL60) cells were used for subsequent experiments. Cells were pretreated with or without SC144 for 1 hour, followed by stimulation with IL-39 for 0 or 3 hours.

For primary cell experiments, mouse bone marrow cells were harvested from femurs and tibias and processed into single-cell suspensions for downstream assays. Cells were then stimulated with rhIL-39 (50 ng/mL) for 0 or 3 h. Following stimulation, cells were collected for total RNA extraction, and the mRNA expression levels of downstream inflammatory signaling molecules were analyzed by quantitative real-time PCR (qRT-PCR). Bone marrow-derived neutrophils (BMDNs) were isolated using the Neutrophil Isolation Kit (Miltenyi Biotec, Cat. No. 130-097-658, Germany) in combination with MACS LS Columns (Miltenyi Biotec, Cat. No. 130-042-401, Germany) according to the manufacturer's instructions. BMDNs and RAW264.7 cells were pretreated with SC144 (2 μM, dissolved in DMSO) or vehicle control (DMSO) for 1 h prior to IL-39 stimulation. The final concentration of DMSO in the culture medium was kept constant at 0.1% (v/v; 1:1000) across all conditions. Cells were harvested at 0 h and 3 h following stimulation for RT-qPCR analysis. For analysis of CXCL1 secretion, cell culture supernatants were collected after IL-39 stimulation and clarified by centrifugation. CXCL1 concentrations were measured using a commercial ELISA kit (Abclonal, RK00038) according to the manufacturer's instructions. For signaling analyses, BMDNs were stimulated with IL-39 (50 ng/mL) for 0, 10, or 30 min in the presence of vehicle or SC144 prior to protein extraction and immunoblotting.

### Protein sequence identity analysis

Full-length amino acid sequences of human and mouse IL23A and EBI3 were retrieved from the UniProt database (Human IL23A: Q9NPF7; Mouse Il23a: Q9EQ14; Human EBI3: Q14213; Mouse Ebi3: O35228) 28. Sequence alignment was performed using the Needleman-Wunsch global alignment algorithm 29. Percent identity was calculated as the number of identical residues divided by the total alignment length (including gap positions).

### Statistical analysis

Data are presented as the mean ± SEM and analyzed using GraphPad Prism software (GraphPad Software, San Diego, USA). A Student's t-test was performed to compare data between two groups. For ≥ 3 groups, one-way ANOVA with appropriate post-hoc tests were used; normality and variance were checked and non-parametric alternatives applied when assumptions were violated. Mantel-Cox log-rank test was employed for the comparison of survival. IL-39 high/low groups were defined by the Youden cut-off from ROC. For all statistical analyses, if *p* < 0.05, the results were considered to be statistically significant (*), values less than 0.01 or 0.001 were shown as ** or ***, respectively.

## Results

### Clinical characteristics of enrolled septic patients and healthy individuals

The cohort comprised 108 septic patients and 56 healthy volunteers admitted to *Shanghai Ruijin Hospital* from 2019 to 2025. For the primary 90-day endpoint (**Table 1**), non-survivors had higher baseline CRP (P = 0.0020), PCT (P = 0.0001), SOFA scores (P = 0.0003), and APACHE II scores (P = 0.0046) than survivors, whereas sex (P = 0.8109), age (P = 0.4022), and WBC count (P = 0.1458) did not differ significantly between the two groups. The 90-day all-cause mortality was 25.0% (27/108).

### IL-39 levels in patients with sepsis are elevated and correlate with the disease severity

**Figure 1:**
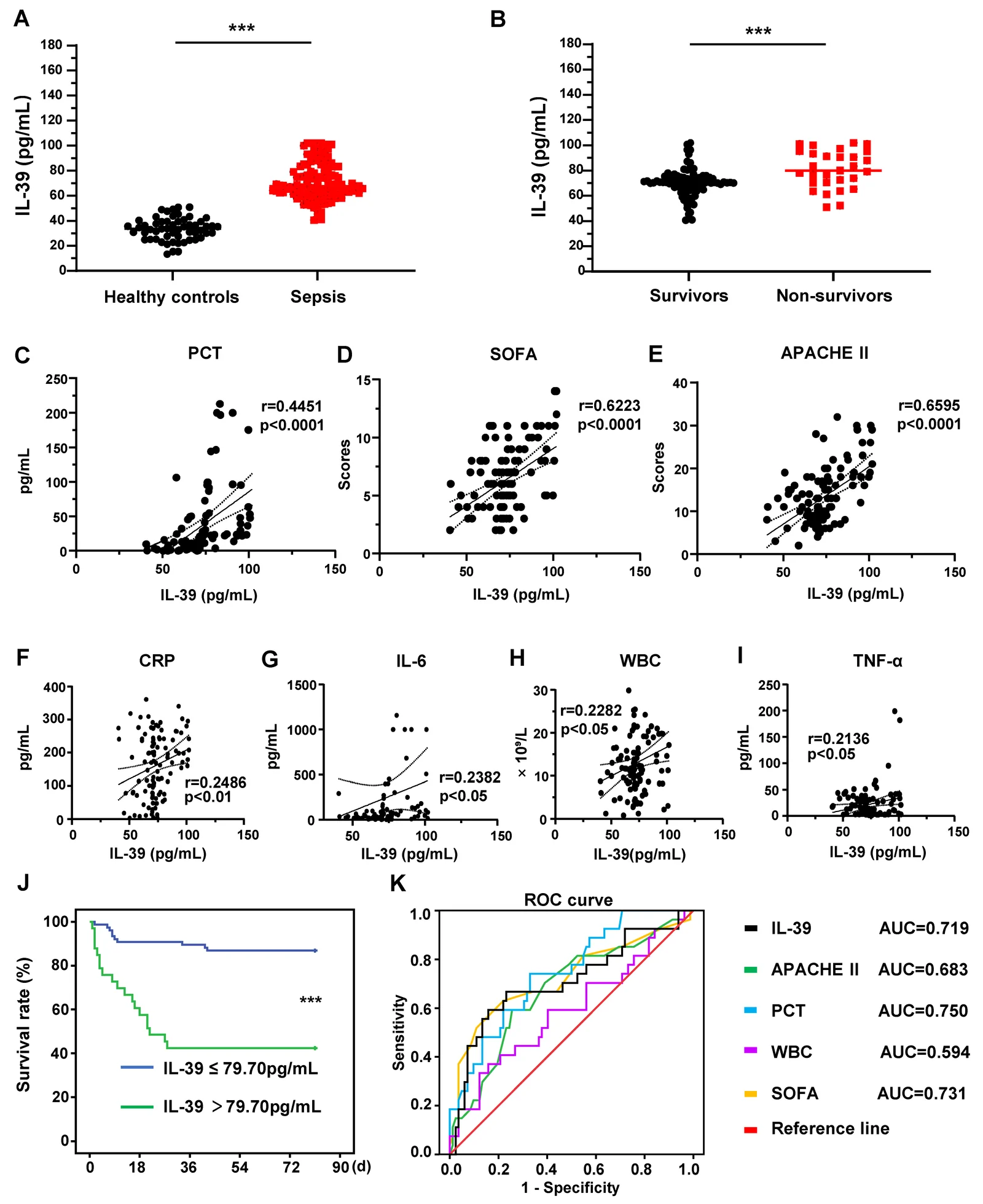
** IL-39 levels are elevated in patients with sepsis, correlating with the severity of the disease.** (**A**) The levels of IL-39 were measured by ELISA in septic patients (n= 108) upon admission and in healthy volunteers (n= 56) with 90-day mortality as the endpoint. Data are presented as median with interquartile range. P value was determined by Mann-Whitney U test. (**B**) The levels of IL-39 in sepsis survivors (n= 81) and non-survivors (n= 27) upon admission with 90-day mortality as the endpoint. Data are presented as median with interquartile range. P value was determined by Mann-Whitney U test. (**C-I**) The relationship between admission IL-39 levels and PCT levels, SOFA scores, APACHE II scores, CRP levels, IL-6 levels, WBC levels and TNF-α levels in septic patients. (**J**) Kaplan-Meier curves by IL-39 cutoff with 90-day mortality as the endpoint. (**K**) ROC curves for predicting 90-day mortality in septic patients using IL-39, SOFA, APACHE II, PCT, and WBC levels at admission.

Serum IL-39 at admission was higher in septic patients than in healthy controls (**Figure 1A**). Among septic patients, 90-day non-survivors had higher baseline IL-39 than survivors (**Figure 1B; Table 1**). From day 0 to day 3, serum IL-39 showed no significant change (Figure S1A). For the supportive 28-day analysis, baseline IL-39 was also higher in non-survivors than in survivors (**Figure S1B**). Furthermore, serum IL-39 levels were positively correlated with PCT, SOFA score, and APACHE II score, and were weakly but significantly correlated with CRP, IL-6, TNF-α, and white blood cell (WBC) count (**Figure 1C-I**). CRRT is widely used in sepsis-associated acute kidney injury, primarily as a supportive strategy for fluid, metabolic, and hemodynamic control. Randomized controlled trials have failed to demonstrate a survival benefit of high-volume compared with standard-dose CRRT in septic shock patients 30. The utilization rate and frequency of CRRT were significantly higher in the non-survivor group. CRRT administration showed no survival benefit in patients at either 28 or 90 days (**Table S1 and Table S5**). Furthermore, stratified analysis was performed for patients according to the IL-39 cutoff value to investigate the impact of CRRT on clinical outcomes. The results demonstrated that CRRT had no effect on the association between IL-39 and clinical outcomes, and the survival difference between the IL-39 High and IL-39 Low groups remained significant after adjusting for CRRT exposure (**Table S8-S9**).

Kaplan-Meier curves stratified by the IL-39 cutoff showed lower 90-day survival in the high-IL-39 group (**Figure 1J**; log-rank P < 0.001). For prediction of 90-day mortality, IL-39 yielded an AUC of 0.719 (95% CI, 0.593-0.845) with a Youden-derived cutoff of 79.70 pg/mL, a sensitivity of 59.3%, and a specificity of 85.2% (P = 0.001; **Table 3**). For comparison, SOFA, APACHE II, PCT, and WBC yielded AUCs of 0.731, 0.683, 0.750, and 0.594, respectively (**Table 3**). In Cox analyses for 90-day mortality, IL-39 was significant in the univariable model (HR 1.057, 95% CI 1.030-1.086; P < 0.0001), but not in the multivariable model including SOFA, APACHE II, PCT, and WBC (HR 1.031, 95% CI 0.985-1.080; P = 0.191; **Table 4**).

As a supportive analysis, 28-day non-survivors showed higher baseline PCT levels (P < 0.0001), SOFA scores (P = 0.0001), and APACHE II scores (P = 0.0453) than survivors, while no significant differences were observed in sex, age, WBC count, and CRP levels between the two groups (all P > 0.05; **Table S5**). For predicting 28-day mortality, IL-39 levels yielded an AUC of 0.751 (95% CI, 0.625-0.876) with a Youden cut-off value of 75.32 pg/mL (a sensitivity of 72.7%, a specificity of 76.7%, P = 0.0001; **Table S6**/**Figure S1D**). Kaplan-Meier curves stratified by the IL-39 cut-off value showed significantly lower 28-day survival rates in the high-IL-39 group (**Figure S1C**; log-rank P < 0.001). In Cox proportional hazards analyses for 28-day mortality, IL-39 emerged as a significant prognostic factor in both univariable Cox analysis (HR 1.065, 95% CI 1.034-1.097; P < 0.0001) and multivariable Cox models (HR 1.057, 95% CI 1.005-1.112; P = 0.032; **Table S7**). Overall, the findings for 28-day endpoint were directionally consistent with those for the 90-day endpoint. Collectively, these data indicate that the levels of IL-39 at admission are elevated in septic patients and are correlated with illness severity. Although IL-39 was not an independent predictor of 90-day mortality in multivariable analysis, it remained significantly associated with 28-day mortality, suggesting its potential as a complementary prognostic marker that warrants further validation.

### Organ-specific SOFA subscores

For all 108 septic patients included in the study, we recorded the SOFA subscores for each organ system at enrollment (respiratory, coagulation, liver, cardiovascular, central nervous system, and renal). We now present these data in **Table S1**, comparing survivors (n = 81) and non-survivors (n = 27) at 90 days.

Briefly, non-survivors had significantly higher serum IL-39 levels at enrollment than survivors (83.54 pg/mL [IQR 65.65-94.22] vs. 66.46 pg/mL [IQR 60.49-74.73], p = 0.00070), as well as higher total SOFA scores (9.00 [5.00-11.00] vs. 5.00 [3.00-7.00], p = 0.00032) and APACHE II scores (18.00 [13.00-20.00] vs. 12.00 [9.00-15.00], p = 0.0046). Among the SOFA organ subscores, liver dysfunction, cardiovascular dysfunction, and central nervous system dysfunction were significantly more severe in non-survivors (p < 0.0001, p < 0.0001, and p = 0.0284, respectively), whereas respiratory, coagulation, and renal subscores did not differ significantly between groups. These findings suggest that poor outcome in our cohort was associated with greater overall illness severity and dysfunction in multiple systems, rather than being attributable to a single dominant organ failure pattern.

### Multivariable adjustment for organ-specific dysfunction

To address the concern that the association between IL-39 and prognosis might be driven by dysfunction of a specific organ system, we performed multivariable logistic regression analyses with 90-day mortality as the dependent variable. IL-39 was modeled as a continuous predictor scaled per 10 pg/mL increase.

In the unadjusted model, higher IL-39 was significantly associated with increased odds of 90-day mortality (OR 1.89 per 10 pg/mL increase; 95% CI 1.35-2.66; p = 0.0002). After simultaneous adjustment for all six SOFA organ subscores, the effect estimates for IL-39 remained positive but was attenuated (adjusted OR 1.41 per 10 pg/mL; 95% CI 0.91-2.19; p = 0.13). We then further adjusted for age, sex, and APACHE II score, and the point estimate for IL-39 remained elevated, although statistical significance was further reduced (adjusted OR 1.31 per 10 pg/mL; 95% CI 0.76-2.27; p = 0.34). In these adjusted models, no individual SOFA organ subscore emerged as an independent predictor.

Together, these analyses show that: (i) IL-39 levels were higher in non-survivors; (ii) non-survivors had greater overall disease severity, with more pronounced liver, cardiovascular, and central nervous system dysfunction; and (iii) the association between IL-39 and 90-day mortality was not attributable to any single SOFA organ subscore alone, although the effect size was attenuated after comprehensive adjustment, likely reflecting overlap with established severity indices and the limited sample size. **Table S1** (baseline IL-39, total SOFA score, and SOFA organ subscores in survivors vs. non-survivors) and **Table S2** (multivariable logistic regression analyses for 90-day mortality) were added.

### IL-39 level is elevated in the mouse model of sepsis induced via cecal ligation and puncture (CLP)

To confirm successful establishment of the CLP model, we monitored rectal temperature of mice every hour after surgery (**Figure S7A**). Peripheral blood levels of alanine aminotransferase (ALT) and aspartate aminotransferase (AST) were measured at 6 h and 8 h after CLP and were significantly elevated (**Figure S7B**). Serum cytokines IFN-γ and IL-6 were also assessed at 6 h and showed marked increases (**Figure S7C**). In addition, lung, liver, kidney, and heart tissues were collected from sham, CLP 6 h, and CLP 8 h mice for organ injury scoring, which revealed significantly aggravated injury in the lung, liver, kidney, and heart following CLP (**Figure S7D**).

**Figure 2:**
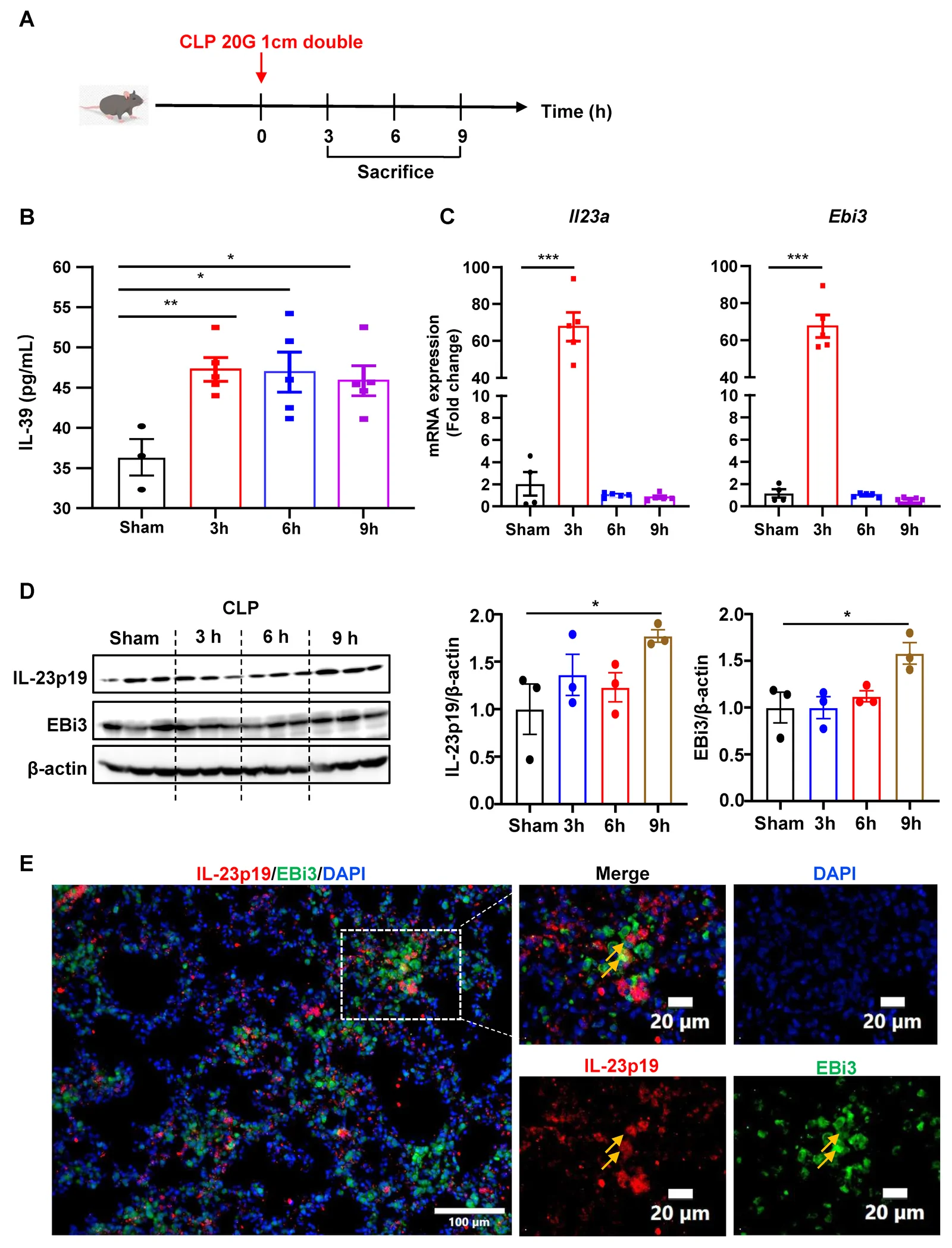
** IL-39 is significantly elevated in cecal ligation and puncture (CLP) induced septic mice compared to sham-operated controls.** (**A**) A schematic timeline of the experimental procedure for constructing the CLP-induced sepsis mouse model. (**B**) ELISA assessment of serum IL-39 levels at various time points in septic mice compared (n= 5 per group) to those in the sham-operated control group (n= 3 per group). (**C**) RT-qPCR analysis of IL-39 gene expression levels (composed of the IL-23A subunit and EBi3 subunit) in mouse spleens at different time points. (**D**) Western blot analysis of IL-39 protein expression levels in the spleens of mice at various time points. (**E**) Immunofluorescence co-staining for IL-23p19 and EBi3 in lung at 14 hours post-CLP to demonstrate their co-localization. The bars represent mean ± SEM.

Based on clinical observations that IL-39 levels are elevated in septic patients and correlate with disease prognosis, suggesting a potential role in disease progression, we aimed to obtain experimental evidence to validate this implication. To verify whether increased levels of IL-39 during sepsis would exacerbate systemic inflammation and disease progression, leading to elevated 90-day mortality, CLP-induced sepsis murine model was used (**Figure 2A**). Serum IL-39 levels were found significantly elevated in septic mice compared to sham-operated controls (**Figure 2B**). Similar to the situation in septic patients, IL-39 levels in septic mice showed sustained elevation throughout the disease course (**Figure 2B**). Subsequently, mice spleen and lung were collected, and the expression levels of IL-39 were evaluated. RT-qPCR analysis indicated a significant upregulation of gene expression of *Il23a* and *Ebi3* in spleen, genes coding for the subunits of IL-39, in septic mice 3 hours post-modeling compared to sham-operated controls (**Figure 2C**). Western blot analysis further demonstrated a remarkable upregulation of IL-39 protein expression levels in septic mice compared to the sham group (**Figure 2D**). We also performed staining of the mouse lungs for the two IL-39 subunits, IL-23p19 and EBi3, and confirmed their co-localization in the septic lung (**Figure 2E**).

### Administration of recombinant human IL-39 (rhIL-39) aggravated disease severity and mortality in CLP-induced septic mice

**Figure 3:**
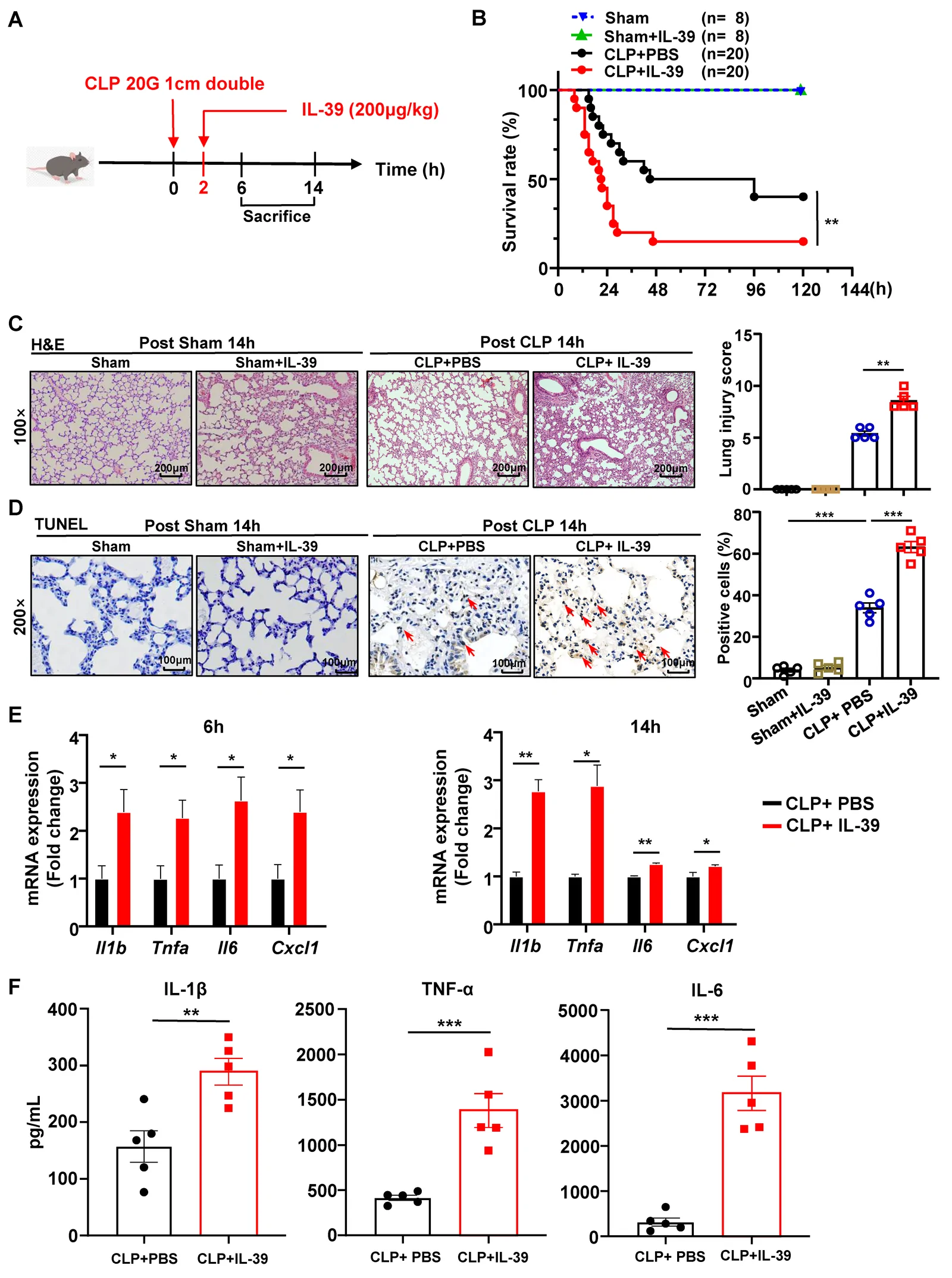
** rhIL-39 exacerbates disease severity in CLP-induced septic mice.** (**A**) Schematic timeline of the experimental procedure for constructing the CLP-induced sepsis mouse model. (**B**) Survival rates of CLP-induced septic mice following rhIL-39 intervention (Sham, n=8; Sham+IL-39, n=8; CLP+PBS, n=20; CLP+IL-39, n= 20). (**C**) Lung tissue HE staining and pathological injury scores in rhIL-39-treated mice compared to controls, 14 hours post-CLP (n= 5 per group). (**D**) TUNEL staining of lung tissues in rhIL-39-treated mice compared to controls, 14 hours post-CLP (n= 5 per group). (**E**) RT-qPCR analysis of mRNA expression levels of inflammatory cytokines in the lungs at 6h and 14h post-CLP. (**F**) ELISA measurement of serum inflammation-related cytokine levels (IL-1β, TNF-α and IL-6) in mice 14 hours after IL-39 intervention (n= 5 per group). The bars represent mean ± SEM.

To verify the hypothesis that IL-39 might exacerbate the severity of sepsis, recombinant human IL-39 (rhIL-39), which is highly homologous to its murine counterpart 19, 31, was administered to CLP-induced septic mice (**Figure 3A**). Full-length amino acid sequences of human and mouse IL23A and EBI3 were retrieved from the UniProt database and aligned using the Needleman-Wunsch global alignment algorithm 28, 29. Percent identity analysis demonstrated that IL23A shares 72.45% and EBI3 shares 60.43% amino acid identity between humans and mice. Administration of a low dose of IL-39 (40 μg/kg) did not affect survival in CLP-induced sepsis (**Figure S4A-B**). In contrast, treatment with a higher dose (200 μg/kg) markedly increased mortality. Based on these results, a dose of 200 μg/kg was used in subsequent experiments. It was observed that the survival rates of rhIL-39 treated 200 μg/kg mice were significantly lower than that of the control group (**Figure 3B**).

In sepsis, lungs are frequently the first to be impacted and are the most susceptible to failure 32. The extent of acute lung injury (ALI) is one of the most critical prognostic factors influencing mortality in patients with sepsis 32. H&E staining revealed that lung injury was markedly more severe in the rhIL-39-treated mice, as indicated by higher injury scores than those in the control group (**Figure 3C**). TUNEL staining of the lung tissues showed a significant increase in apoptotic cells in the rhIL-39-treated mice compared to controls (**Figure 3D**). In contrast, rhIL-39 administration did not alter survival, lung histology or apoptosis in sham-operated mice (**Figure 3B, 3C and 3D**)**.** Additionally, RT-qPCR analysis of mRNA expression of inflammatory cytokine in the lungs demonstrated a notable upregulation of inflammatory markers in the rhIL-39-treated mice, including *Il1b*, *Tnfa*, *Il6* and *Cxcl1* (**Figure 3E**). To validate the obtained RT-qPCR results, serum samples were collected from mice 14 hours after CLP modeling, and ELISA was performed to measure the pro-inflammatory cytokines (IL-1β, TNF-α, and IL-6). As shown in **Figure 3F**, the serum levels of pro-inflammatory cytokines in rhIL-39-treated mice were significantly upregulated when compared to the control mice. Thus, these findings suggest that administration of rhIL-39 exacerbates the severity of sepsis in mice.

### Administration of IL-39 neutralizing antibodies ameliorated disease severity in CLP-induced septic mice

**Figure 4:**
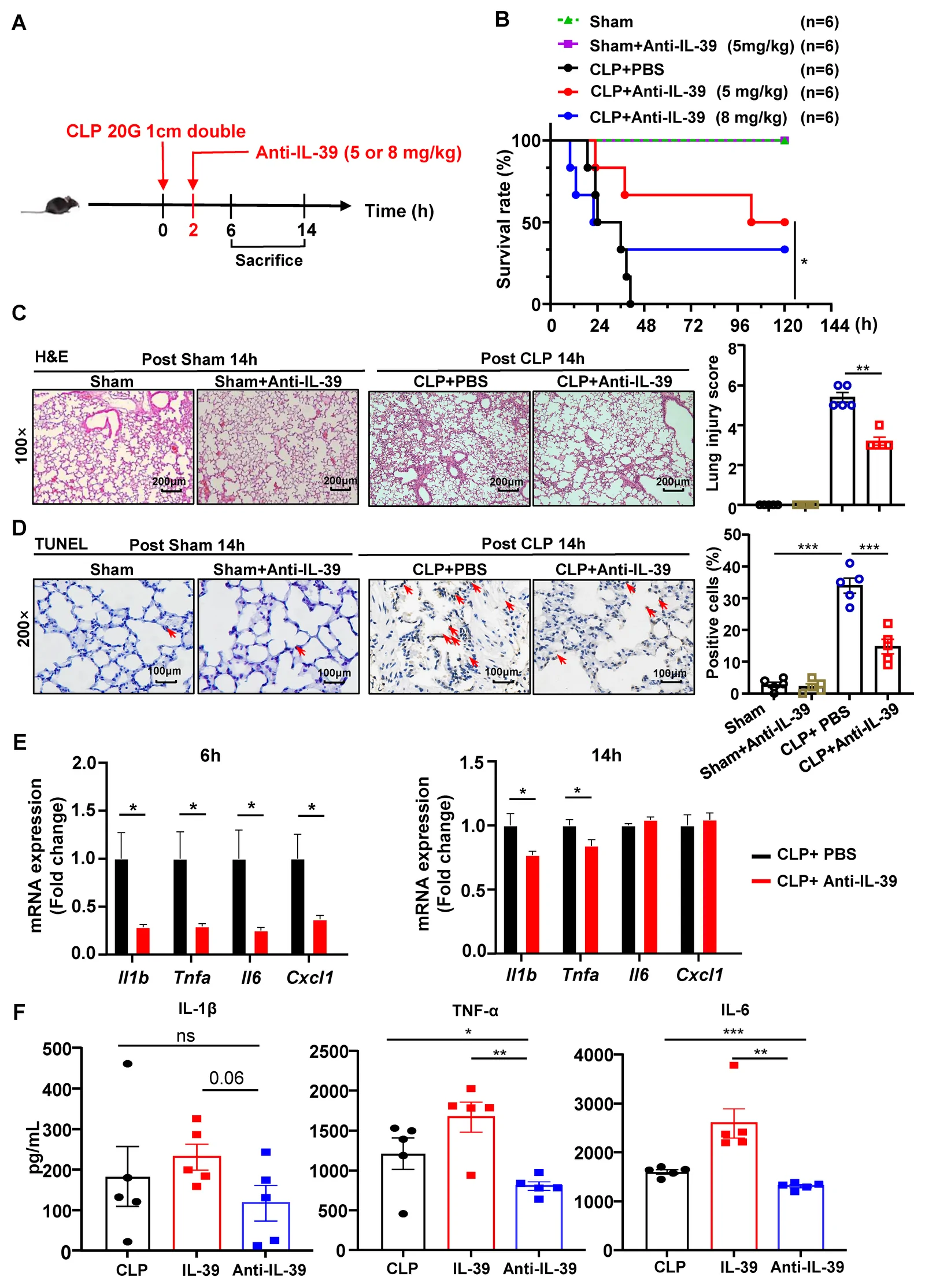
** IL-39 neutralizing antibodies alleviate disease severity in CLP-induced septic mice.** (**A**) Schematic timeline of the experimental procedure for constructing the CLP-induced sepsis mouse model. (**B**) Anti-IL-39 at 5 mg/kg significantly reduced mortality in the CLP-induced sepsis mouse model, whereas the higher dose of 8 mg/kg failed to confer protection. (n= 6 per group). (**C**) Lung tissue HE staining and pathological injury scores in neutralizing antibodies treated mice compared to controls, 14 hours post-CLP (n= 5 per group). (**D**) TUNEL staining of lung tissues in neutralizing antibodies treated mice compared to controls, 14 hours post-CLP (n= 5 per group). (**E**) RT-qPCR analysis of mRNA expression levels of inflammatory cytokines in the lungs at 6- and 14-hours post-CLP. (**F**) ELISA measurement of lung tissue inflammation-related cytokine levels (IL-1β, TNF-α and IL-6) in mice 14 hours after anti-IL-39 intervention (n= 5 per group). The bars represent mean ± SEM.

To further validate the role of IL-39 in sepsis, IL-39 neutralizing antibodies were injected intraperitoneally into CLP-induced septic mice as therapeutic intervention (**Figure 4A**). Mice treated with 5mg/kg IL-39 neutralizing antibodies exhibited a significant improvement in survival compared with the control group (**Figure 4B**). However, no additional survival benefits were observed at the lower dose of 4mg/kg (**Figure S2C-D**) or higher dose of 8mg/kg (**Figure 4B**). IL-39 is a heterodimeric cytokine consisting of the IL-23p19 and EBI3 subunits. Notably, neutralization of the IL-23p19 subunit conferred a significant survival benefit, whereas anti-EBI3 was ineffective, performing no better than the control (**Figure S2F and Figure S2G**). Histopathological analysis with H&E staining revealed significantly lower lung injury scores in IL-39 antibody-treated mice (**Figure 4C**). TUNEL staining further demonstrated a reduction in apoptotic cells in the lungs of IL-39 neutralizing antibodies treated mice (**Figure 4D**). Notably, administration of anti-IL-39 antibody did not alter survival, lung histopathology or apoptosis in sham-operated mice (**Figure 4B, 4C and 4D**). Although ELISA analysis of serum inflammatory cytokines (IL-1β, TNF-α, and IL-6) in IL-39 antibody-treated mice showed no significant differences compared to control mice, as demonstrated in **Figure S3A**, RT-qPCR analysis of lung tissue revealed a marked downregulation in mRNA expression levels of pro-inflammatory cytokines following IL-39 neutralizing antibody therapy (**Figure 4E**). The levels of IL-1β, TNF-α, and IL-6 in homogenates of lung tissues were significantly lower in IL-39 antibody-treated group compared with the IL-39 group (**Figure 4F**). In spleen tissues, IL-1β and TNF-α were also markedly reduced in the IL-39 neutralizing antibody group (**Figure S3B**). Collectively, these findings suggest that IL-39 neutralizing antibodies administered at an appropriate concentration can effectively alleviate the severity of sepsis in CLP-induced septic mice.

### IL-39 increases the mortality of CLP-induced septic mice by promoting neutrophil infiltration

**Figure 5:**
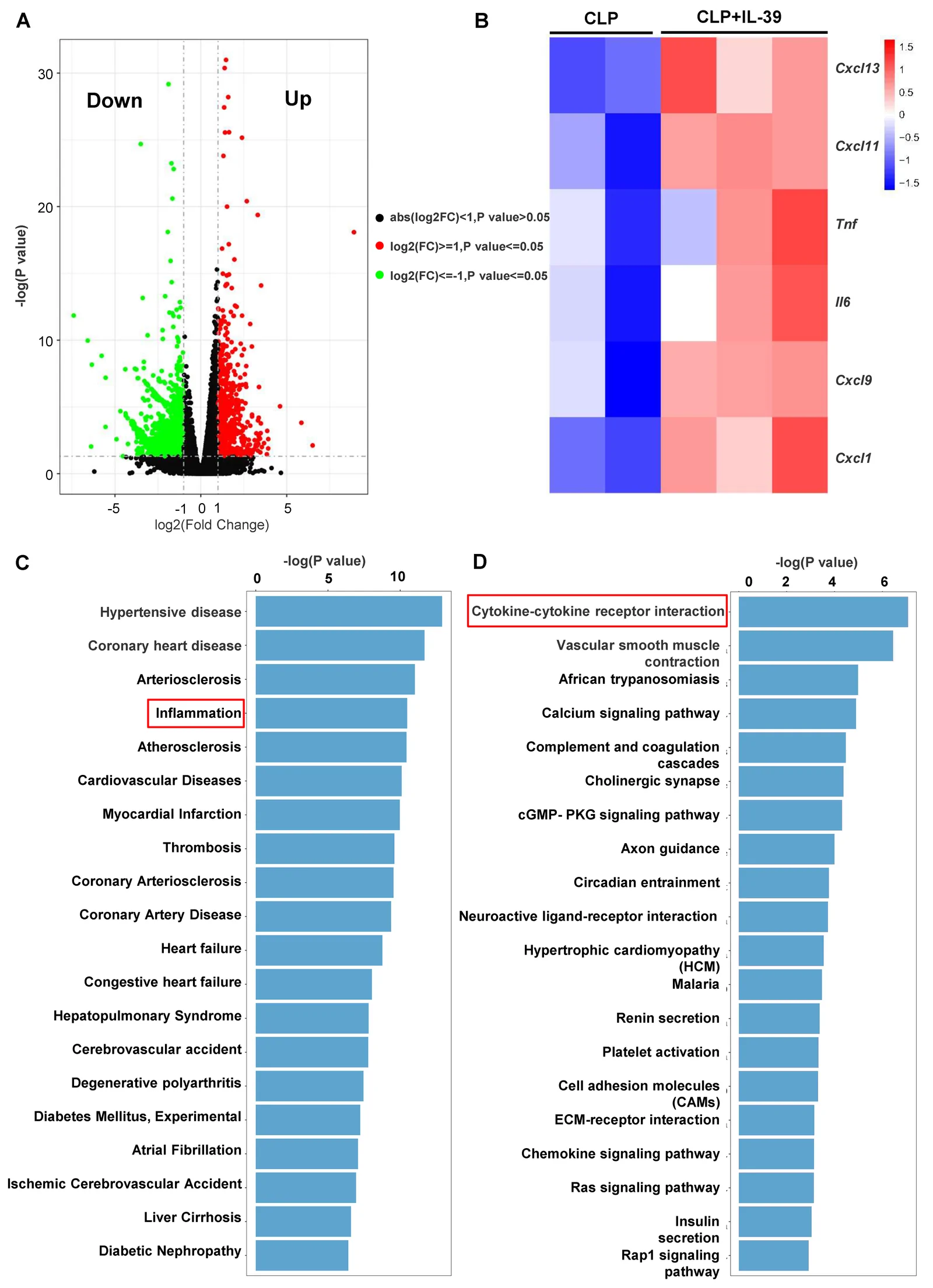
** Transcriptomic analysis of lung/spleen tissues from CLP mice following IL-39 intervention.** (**A**) Volcano plot depicting differentially expressed genes (DEGs), defined as genes with |log2(fold change)| ≥ 1 and adjusted P value (Benjamini-Hochberg FDR) < 0.05, identified by RNA sequencing. (**B**) Upregulation of key chemokines and pro-inflammatory cytokines among the DEGs. (**C**) KEGG disease enrichment analysis showing association of DEGs with inflammatory diseases. (**D**) Pathway enrichment analysis revealing significant enrichment of DEGs in the Cytokine-cytokine receptor interaction pathway.

**Figure 6:**
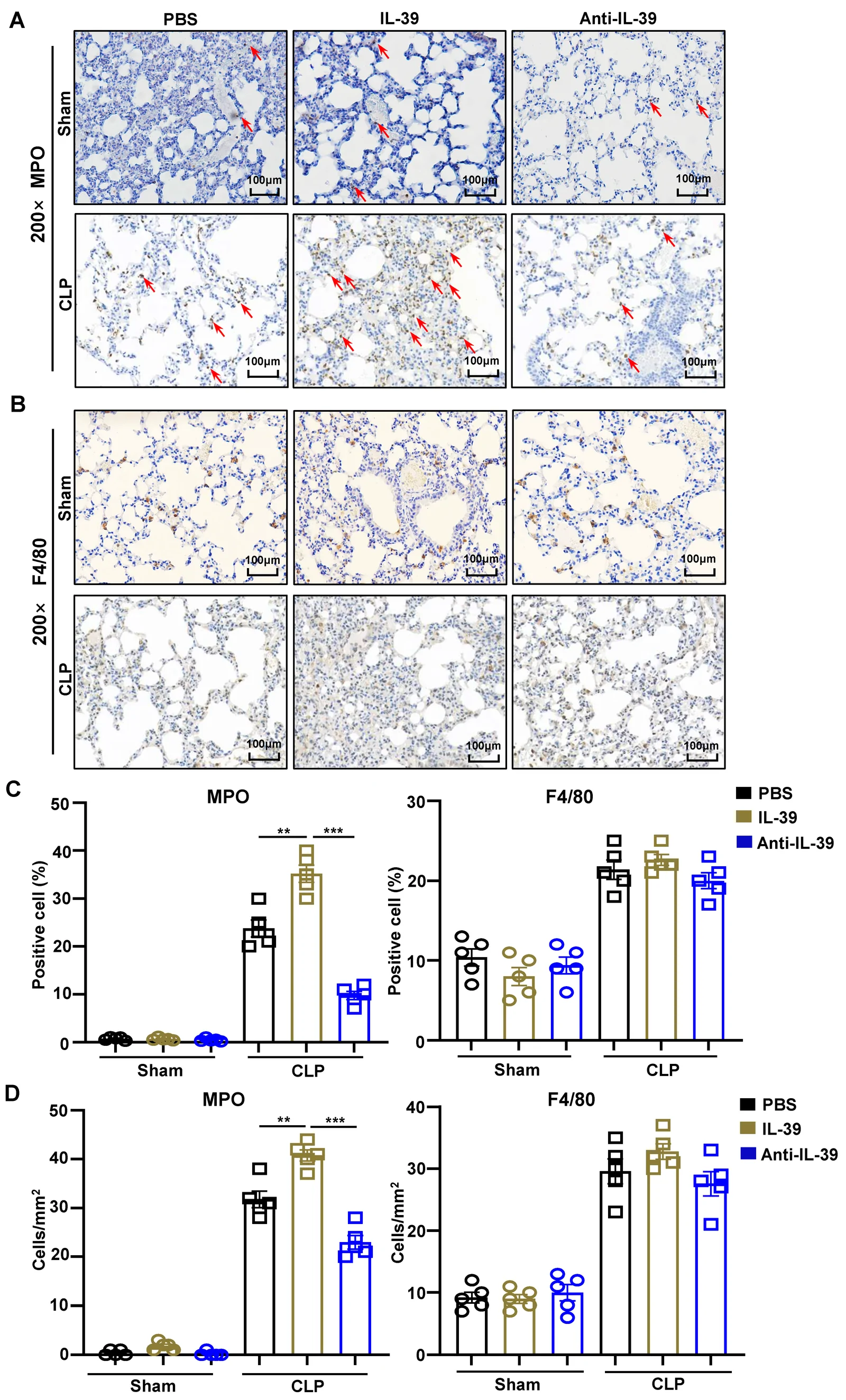
** IL-39 regulates pulmonary inflammatory responses in murine sepsis.** (**A**) Representative myeloperoxidase (MPO) staining of lung sections from mice 14 hours after CLP, showing three groups: PBS (n= 5 per group), IL-39 (n= 5 per group) and anti-IL-39 (n= 5 per group). (**B**) Representative F4/80 staining of lung sections from mice 14 hours after CLP, showing three groups: PBS (n= 5 per group), IL-39 (n= 5 per group) and anti-IL-39 (n= 5 per group). (**C**) Quantification of the percentage of MPO or F4/80 positive cells in lung sections. (**D**) Quantification of MPO or F4/80 positive cells in lung sections using three complementary metrics-percentage among DAPI⁺ nuclei per HPF, density (cells/mm²), and positive area fraction—averaged over five non-overlapping HPFs per mouse (ImageJ workflow; analyst blinded). The bars represent mean ± SEM.

To investigate the systemic immunological changes in CLP mice following IL-39 intervention, we performed RNA sequencing (RNA-seq) on the lung tissues. **Figure 5A** displays the volcano plot of identified differentially expressed genes (DEGs). The upregulation of several genes encoding critical chemokines and pro-inflammatory cytokines, such as *Il6*, *Tnfa*, *Cxcl1*, and *Cxcl9*, was a hallmark of the transcriptomic response, suggesting a potent activation of innate immune signaling pathways. (**Figure 5B**). KEGG disease enrichment analysis further indicated a correlation with inflammatory diseases (**Figure 5C**). Furthermore, pathway enrichment analysis demonstrated that the DEGs were highly enriched in the "Cytokine-cytokine receptor interaction" pathway (**Figure 5D**). Previous studies have demonstrated that neutrophils and macrophages play a pivotal role in acute respiratory distress syndrome (ARDS) caused by sepsis 33, 34. Therefore, we performed immunohistochemical analyses on lung tissues of CLP-induced septic mice. In sham-operated mice, MPO and F4/80 staining was minimal, and treatment with either rhIL-39 or anti-IL-39 did not significantly alter these baseline levels (**Figure 6A**). In contrast, rhIL-39 administration markedly increased the proportion of neutrophils, whereas IL-39-neutralizing antibody treatment substantially reduced their relative frequency compared with the corresponding control groups (**Figure 6A, C**). In contrast, no substantial alterations were found in macrophage numbers (**Figure 6B-C**).

**Figure 7:**
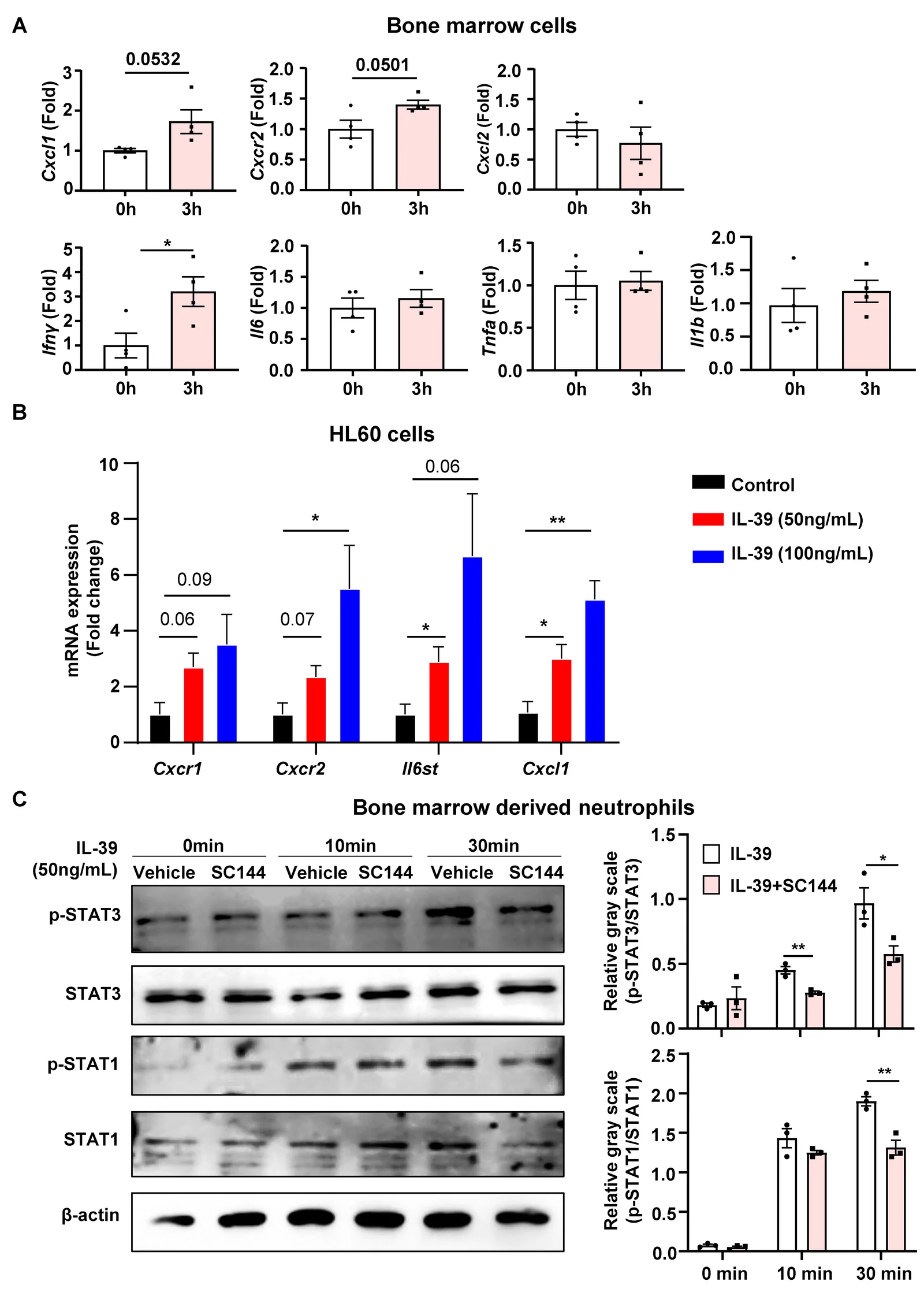
** IL-39 promotes neutrophil recruitment by upregulating CXCL1 expression.** (**A**) IL-39 (50 ng/mL) stimulation of bone marrow cells upregulated the expression of Cxcl1 and Cxcr2. Although these increases did not reach statistical significance (P = 0.0532 and 0.0501, respectively), Ifnγ expression was significantly elevated (P < 0.05). (**B**) IL-39 at 50 ng/mL or 100 ng/mL significantly upregulated the expression of Cxcr2, Il6st, and Cxcl1 in neutrophils. (**C**) Western-blot results showed that treatment with the IL-6st inhibitor SC144 significantly suppressed IL-39-induced phosphorylation of STAT1 and STAT3. The bars represent mean ± SEM.

Quantification of cell density revealed that IL-39 treatment significantly increased neutrophil infiltration, which was attenuated by treatment with an IL-39 antibody. In contrast, IL-39 treatment did not enhance macrophage infiltration **(Figure 6D)**. Previous investigations have demonstrated that IL-39 exerts its biological effects through the IL-23 receptor (IL-23R) and the IL-6 signal transducer (IL-6ST, also known as GP130) 18, 31. In order to confirm the expression of IL-39-related receptors on neutrophils in septic patients, we analyzed publicly available single-cell datasets via Single Cell Portal and found that IL-6ST is expressed in septic patients, with its localization specifically detected on neutrophils (**Figure S4A**) 35. This suggests that IL-39 might exert its effects *via* IL-6ST on the neutrophil surface. Accordingly, we treated mouse bone marrow cells with IL-39 and found that although *Cxcl1* and *Cxcr2* transcript levels were mildly increased, these changes did not reach statistical significance. In contrast, IL-39 induced a robust and significant upregulation of *Ifnγ* expression, consistent with previous reports** (Figure 7A)**. Subsequently, we performed additional *in vitro* experiments using the HL60 cells (a neutrophil cell line) and RAW264.7 cells (a macrophage cell line) to further determine the potential influence of IL-39 on immune cells. RT-qPCR analyses showed that IL-39 did not increase the expression of inflammatory factors in RAW264.7 macrophages (**Figure S4B**). In contrast, although dHL60 cells showed no detectable elevation in inflammation-related cytokines following IL-39 treatment (**Figure S4C**), they exhibited significant upregulation of the neutrophil chemokine *Cxc1*, the C-X-C motif chemokine receptor 1 (*Cxcr1*), the C-X-C motif chemokine receptor 2 (*Cxcr2*), and the IL-39-associated receptor *Il6st* (**Figure 7B**). Further analysis of IL-39 downstream signaling revealed concurrent activation of the p-STAT1 and p-STAT3 pathways. Notably, this effect was abolished by SC144, an IL-6ST inhibitor, confirming the involvement of IL-6ST in IL-39 signaling (**Figure 7C**).

**Figure 8:**
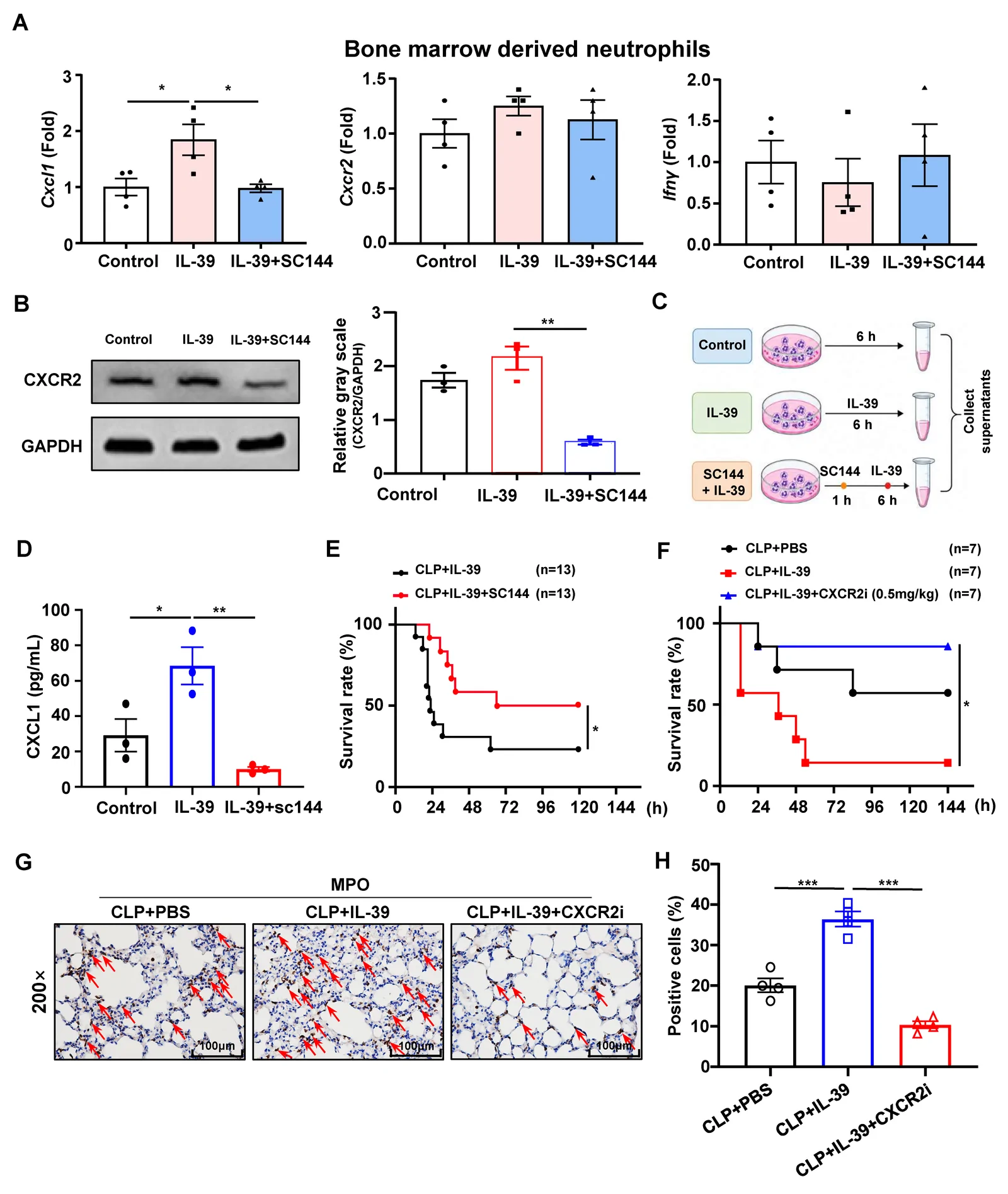
** Inhibition of the IL-39-CXCL1-CXCR2 signaling pathway significantly alleviates IL-39-exacerbated mortality in CLP mice.** (**A**) SC144 treatment significantly suppressed IL-39-induced upregulation of *Cxcl1*. (**B**) SC144 administration effectively attenuated IL-39-induced upregulation of CXCR2 protein expression. (**C**) Schematic timeline illustrating the experimental procedure for CXCL1 protein detection in culture supernatants from primary bone marrow-derived neutrophils (BMDNs) in the Control, IL-39 and SC144+IL-39 groups. (**D**) ELISA measurement of CXCL1 levels in culture supernatants from primary BMDNs in the Control, IL-39, and SC144+IL-39 groups. (**E**) SC144 treatment markedly alleviated the increased mortality induced by IL-39 in mice (n= 13 per group). (**F**) Administration of CXCR2 inhibitor (CXCR2i) (0.5mg/kg) markedly reduced IL-39-exacerbated mortality in mice (n= 7 per group). (**G**) Representative images of myeloperoxidase (MPO) staining in lung sections from mice in CLP+PBS, CLP+IL-39, and CLP+IL-39+CXCR2i groups. (H) Quantification of the percentage of MPO positive cells in lung sections. Data are presented as mean ± SEM.

Furthermore, treatment with SC144 also suppressed the IL-39-induced upregulation of *Cxcl1* mRNA and CXCR2 protein levels (**Figure 8A and Figure 8B**). Consistently, ELISA measurement of culture supernatants from mouse primary bone marrow derived neutrophils revealed that IL-39 significantly increased CXCL1 protein secretion, whereas SC144 pretreatment markedly attenuated this effect (**Figure 8C and 8D**). Previous evidence suggests that elevated expression of neutrophil chemokines leads to an increased infiltration of neutrophils, which in turn exacerbates tissue damage in ARDS-related sepsis 36, 37. To further validate the role of the IL-6ST receptor in IL-39-mediated inflammation, CLP mice were administered with IL-39 and the IL-6ST inhibitor SC144 via intraperitoneal injection, and subsequent survival rates were monitored. As depicted in **Figure 8E**, intraperitoneal administration of IL-39 combined with SC144 partially improved the survival rate of CLP mice compared to the IL-39 treatment group.

CXCR2 has been verified to play a critical role in the development of early-stage acute respiratory distress syndrome (ARDS) during sepsis, and that this effect is closely linked to neutrophil activity 37. In subsequent experiments using CLP mice, administration of the CXCR2 inhibitor at 0.5 mg/kg significantly improved the mortality exacerbated by IL-39 (**Figure 8F**), whereas a higher dose of 2 mg/kg unexpectedly worsened outcomes (**Figure S5A-B**), indicating that neutrophil infiltration must be modulated within an optimal range. Consistently, MPO staining of lung sections showed that CXCR2 inhibition significantly attenuated IL-39-induced neutrophil infiltration in CLP mice (**Figure 8G and 8H**). These results indicate that IL-39 treatment induces a marked increase in CXCR2 expression compared to the control, however, an effect that is abrogated when IL-39 is co- administered with SC144. Taken together, the *in vivo* and *in vitro* data demonstrate that IL-39 enhances the mortality in CLP-induced septic mice by promoting neutrophil infiltration, which can be effectively counteracted through IL-39 neutralizing strategies.

## Discussion

**Figure 9:**
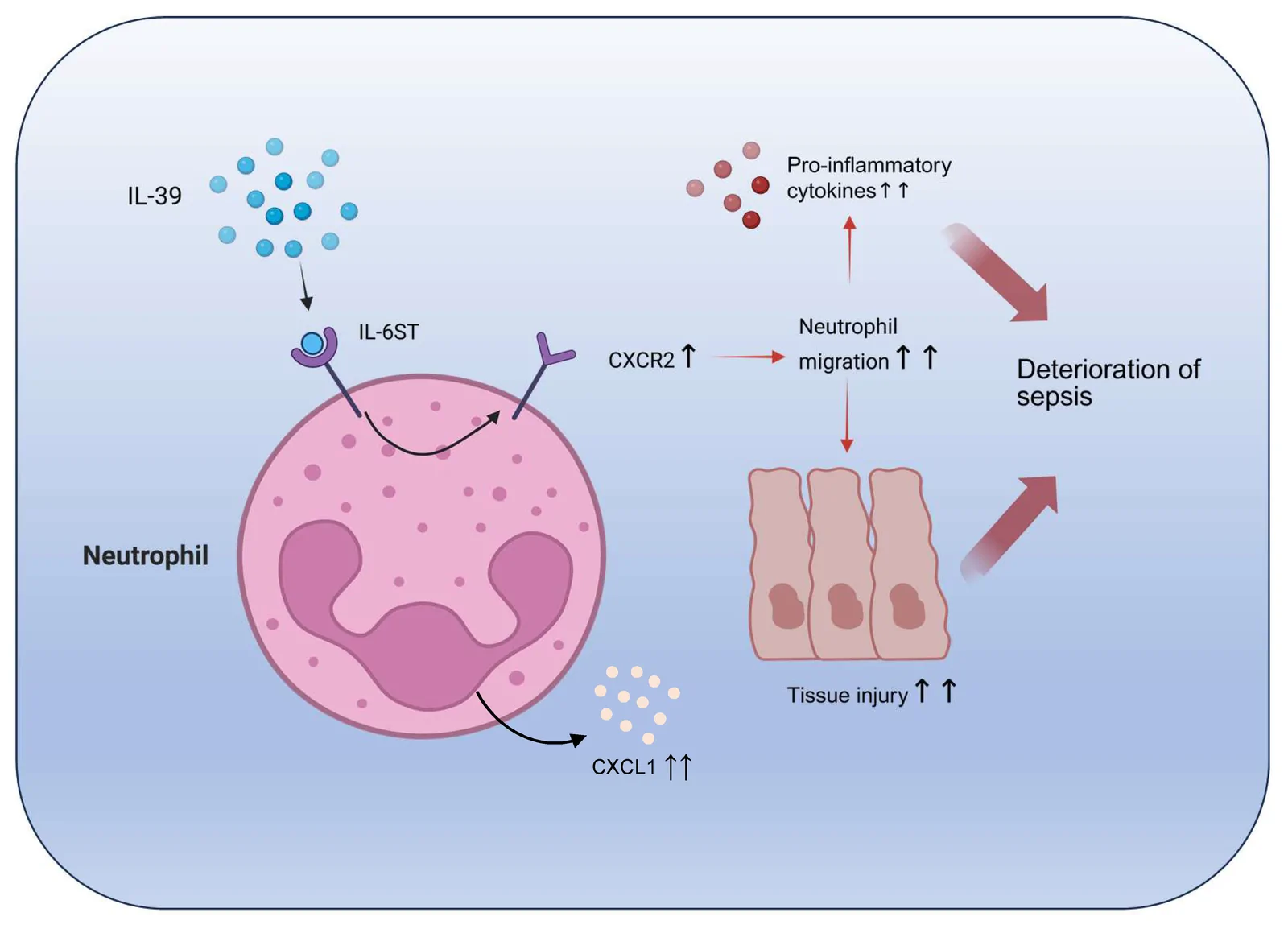
** Proposed working model for the role of IL-39 in sepsis.** The novel cytokine IL-39, primarily secreted by B cells 18, 84, appears to accelerate sepsis progression by targeting neutrophils through binding to the receptor IL-6ST. This ligand-receptor interaction activates the CXCL1-CXCR2 signaling pathway, thereby promoting neutrophil recruitment and infiltration into pulmonary tissues. The resulting chemotactic response enhances the release of pro-inflammatory mediators, amplifying immune-driven tissue injury and further aggravating the pathological cascade of sepsis (This figure was created in https://BioRender.com).

This study reports for the first time the role of cytokine IL-39 in sepsis progression. We found that serum levels of IL-39 in patients with clinical sepsis were significantly higher than those in healthy controls, and a positive correlation was found between the level of IL-39 and mortality. Elevated levels of IL-39 were detected in CLP-induced septic mice as well. Administration of rhIL-39 to these mice led to an increased mortality rate. In contrast, treatment with IL-39 neutralizing antibodies led to a reduction of mortality. Beyond the original 90-day endpoint, we additionally analyzed 28-day survival and observed a trend toward improved survival in the low-IL-39 group, supporting the clinical relevance of IL-39 across early and longer-term horizons. We also explored CRRT as a covariate; while the present study centers on IL-39 biology, the impact of CRRT on outcomes warrants evaluation in larger, standardized cohorts. In addition, the present study provides evidence indicating that neutrophils play a pivotal role in IL-39-mediated inflammatory injury in sepsis *via* the modulation of the CXCL1-CXCR2 pathway (**Figure 9**).

CRRT is widely used in sepsis-associated acute kidney injury, primarily as a supportive strategy for fluid, metabolic, and hemodynamic control. Randomized controlled trials have failed to demonstrate a survival benefit of high-volume compared with standard-dose CRRT in septic shock patients 30. Although early initiation of CRRT may improve short-term outcomes in selected populations, consistent benefits in long-term survival or renal recovery have not been confirmed 38, 39. Moreover, adjunctive cytokine or endotoxin adsorption strategies combined with CRRT show heterogeneous results with limited and low-certainty evidence 40. In the present study, CRRT administration neither conferred survival benefit at 28 or 90 days nor affected the association between IL-39 and clinical outcomes, consisting with the conclusions reported in the existing literature.

Sepsis is a global health issue, representing one of the leading causes of death in ICUs 10, 41. In clinical practice, the early identification of sepsis is of great importance, as timely recognition and treatment can significantly improve survival rates 42. However, no specific biomarker exists currently for the early identification and prognostic assessment of sepsis 43. Early prognostic evaluation of sepsis enables clinicians to predict disease risk and improve clinical care and treatment for septic patients 43-46. Our findings indicate that the levels of IL-39 are significantly elevated in septic patients, with supportive trends at 28 days, suggesting that IL-39 might serve as a potential biomarker for early identification and prognostic assessment of sepsis in clinical practice. Regarding baseline severity, SOFA and APACHE II behaved in the expected direction in our analysis (higher in non-survivors), with acceptable discrimination for mortality. We acknowledge that some datasets may observe attenuated group differences; plausible explanations include limited statistical power, timing variability of score acquisition, heterogeneity in comorbidities, and treatment regimens (e.g., vasopressors, ventilation, CRRT). In our multivariable Cox models adjusted for SOFA and APACHE II, the direction of association between IL-39 and outcomes remained consistent with the primary observation. In the present study, elevated IL-39 levels were positively associated with poor prognosis in sepsis across different analytical settings. These findings suggest that IL-39 may offer complementary prognostic information in certain contexts, though it is not an independent predictor of 90-day mortality beyond SOFA, APACHE II, PCT, and WBC, while its association with 28-day mortality remained significant after adjustment. However, further validation in larger, multi-center cohorts is still warranted to determine whether IL-39 can be firmly established as an independent prognostic marker and to clarify its value for risk stratification and clinical decision-making. As shown in Figure 1B, IL-39 levels were significantly higher in non-survivors, although overlap between the two groups was observed. This variability is not unexpected given the recognized heterogeneity of sepsis 47, 48, and similar overlap has been reported for other inflammatory biomarkers in heterogeneous conditions 49. These observations suggest that IL-39 is best evaluated as part of a broader biomarker panel and in the context of clinical severity. Its potential clinical relevance may reside in its capacity to reflect a specific inflammatory pathway, and its role in risk stratification warrants further prospective evaluation. In the present cohort, IL-39 demonstrated significant discriminatory value for predicting 90-day mortality in sepsis, with an AUC of 0.719 and a specificity of 85.2%. While the sensitivity was moderate, these findings suggest that IL-39 may have potential utility as a prognostic biomarker, particularly for identifying patients at higher risk of poor outcomes. Its predictive performance was also broadly comparable to that of established severity scores, such as SOFA and APACHE II. Nevertheless, further multicenter validation is warranted to clarify its role in clinical risk stratification. This suggests, therefore, that IL-39 might serve as a potential biomarker for further evaluation in sepsis prognosis, particularly in combination with established severity scores. Notably, anti-IL-39 treatment exhibited a bell-shaped (hormetic) dose-response relationship: the intermediate dose (5mg/kg) conferred maximal benefit, whereas 4mg/kg and 8mg/kg did not, consistent with immunopharmacologic non-linearity driven by feedback regulation, receptor saturation or desensitization, and compensatory loops 50. Such non-linear or biphasic response patterns are widely recognized in immunomodulatory and cytokine-targeted therapies and reflect the complex homeostatic regulation of immune signaling pathways 51, 52. Similar “window-of-efficacy” phenomena have been described for other immunomodulatory therapies in sepsis models 51. At lower doses, the circulating levels of the IL-39 neutralizing antibody might be insufficient to achieve complete IL-39 blockade, resulting in only partial suppression of downstream gp130-associated inflammatory signaling. Consequently, residual cytokine signaling may sustain systemic inflammation and ongoing tissue injury. In contrast, higher antibody doses may trigger compensatory immune feedback mechanisms, receptor desensitization, or even paradoxical pro-inflammatory effects, as reported in other cytokine-modulating interventions 53, 54. Excessive cytokine blockade may also disturb physiological immune responses required for effective pathogen clearance, thereby inadvertently exacerbating systemic inflammation or promoting off-target organ damage. No overt acute toxicity was observed at the effective dose, but the narrow therapeutic window warrants careful safety monitoring in future development.

Sepsis is a clinical condition associated with high mortality. One of the primary methods for evaluating the treatment efficacy is through the assessment of changes in 28-day or 90-day survival rates 43, 55. The high early mortality of sepsis is closely correlated with systemic inflammatory response induced by cytokine storm 5, 56. As a novel cytokine, the pro-inflammatory effects of IL-39 have been preliminarily studied and confirmed in conditions like SLE and GVHD 18, 19. However, its role in other inflammatory diseases remains unclear. In the present study, we established the classical CLP-induced sepsis mouse model 22 and performed interventions to evaluate the potential impact of IL-39 on sepsis. The validity of the CLP-induced sepsis model was further supported by dynamic physiological, biochemical, inflammatory, and histopathological changes after surgery, including progressive hypothermia, elevated serum ALT and AST, increased circulating inflammatory cytokines, and significant injury in the lung, liver, kidney, and heart. In addition, the use of rhIL-39 in the murine model was supported by the substantial sequence conservation of its two subunits, IL23A and EBI3, between humans and mice. Full-length sequence comparison showed that IL23A and EBI3 share 72.45% and 60.43% amino acid identity, respectively, supporting the biological plausibility of cross-species activity, although not proving complete functional equivalence 28, 29. We found that rhIL-39 administration to septic mice significantly reduced their survival rate. In contrast, treatment with IL-39 neutralizing antibodies effectively improved the survival rate of CLP mice, suggesting that IL-39, as a pro-inflammatory factor, might contribute to the exacerbation of sepsis. In addition, the specificity of the protective effect requires cautious interpretation. Neutralization of IL-23p19, but not EBI3, produced a survival benefit similar to that of anti-IL-39, suggesting that the observed efficacy may partially reflect suppression of p19-dependent inflammatory signaling, including IL-23-mediated pathways, rather than IL-39 blockade alone. However, the lack of benefit with anti-EBI3 does not support a non-specific generalized inhibition of all IL-12 family cytokines. Thus, while these results reinforce the pathogenic relevance of p19-related inflammation in sepsis, the precise distinction between IL-39-specific and IL-23-dependent effects will require further study using more selective experimental strategies 57, 58.

In septic mice, the levels of pro-inflammatory cytokine (IL-1β, TNF-α, and IL-6) were significantly decreased in the lung and spleen, but not in the serum, following treatment of the IL-39 neutralizing antibody. These findings suggest that IL-39 may serve as a mechanistic link between sepsis and the induction of local inflammation in target organs. Accordingly, inhibition of IL-39 signaling may attenuate sepsis progression by suppressing local pro-inflammatory cytokine production within target organs.

Sepsis is often accompanied by life-threatening multi-organ failure, and the lungs are the first and most frequently affected organ 59. The associated ARDS, or acute lung injury (ALI), is one of the most critical prognostic factors for mortality in septic patients. Improving ARDS is crucial for enhancing the prognosis of sepsis 21, 32. Considering that the current clinical strategy for relieving lung injury induced by sepsis mainly relies on supportive ventilation 32, 33, our findings provide evidence regarding the potential therapeutic value of IL-39 and its related antibodies in mitigating ARDS caused by sepsis, thereby offering a basis for future clinical investigations. This is consistent with the existing findings reporting the fact that IL-39 neutralizing antibodies could improve inflammatory organ damage in diseases like GVHD and SLE 19, 60.

During the acute phase of infection, neutrophils are indispensable in host defense 61. They eliminate pathogens through phagocytosis and the deployment of neutrophil extracellular traps (NETs) 37, 62, 63. However, while these mechanisms are critical for pathogen clearance, they can simultaneously induce cytotoxic effects on host tissues and cells, thereby exacerbating inflammation and leading to organ damage 64. Therefore, mitigating the cytotoxic effects of neutrophils constitutes a crucial strategy in the treatment of sepsis. Within the extensive chemokine superfamily, the CXCR2 receptor emerges as a pluripotent mediator exhibiting heterogeneous cellular expression patterns, with notable prevalence in neutrophil populations 65, 66. Empirical studies demonstrate the regulatory dominance of CXCR2 during early-phase neutrophilic hyperactivation and subsequent tissue injury progression in septic conditions 37, 67. Current evidence suggests that pulmonary endothelial cells produce substantial amounts of CXCL1 during the acute inflammatory response in ARDS 68. Our investigation demonstrates that IL-39 significantly enhances pulmonary neutrophil infiltration in CLP-induced septic mice, an effect mediated through neutrophil CXCL1-CXCR2 pathway activation. Pharmacological inhibition of CXCR2 substantially reversed the survival disadvantage induced by IL-39 in CLP mice, providing direct in vivo functional evidence that activation of the CXCL1-CXCR2 pathway is an important mechanism by which IL-39 aggravates sepsis. These findings provide compelling evidence for the pro-inflammatory role of this novel cytokine in the pathogenesis of sepsis while establishing a mechanistic link between IL-39-mediated responses and canonical septic pathways. In 2021, Nascimento et al. demonstrated that sepsis expands a CD39⁺ plasmablast population, which promotes the adenosine-mediated suppression of macrophage antimicrobial activity, thereby contributing to immune paralysis 4. While our study highlights an IL-39-associated pro-inflammatory signature and its links to disease severity and outcomes, integrating these findings suggests a balanced immune regulatory network in sepsis, with a pro-inflammatory activation driven by IL-39 on one side and CD39⁺ B cells/adenosine-mediated counter-regulation on the other. Conceptually, IL-39 produced under inflammatory conditions could coexist with, or even precede, the emergence of CD39⁺ B-cell responses that suppress host defense, although our study did not directly investigate this axis. Our future studies should investigate whether circulating IL-39 correlates with B-cell/plasmablast phenotypes and CD39/adenosine signatures and whether modulating IL-39 alters macrophage function in the presence of CD39⁺ plasmablasts. This paradigm-shifting discovery not only expands our understanding of cytokine networks in systemic inflammation, but also proposes an innovative therapeutic strategy targeting IL-39 signaling pathway for the management of sepsis, potentially addressing current limitations in clinical interventions.

Although we did not perform murine receptor-binding assays, we verified the biological activity of rhIL-39 in murine cells through STAT signaling activation experiments. Specifically, rhIL-39 significantly induced phosphorylation of STAT1 and STAT3 (p-STAT1 and p-STAT3) in murine bone marrow derived neutrophils, confirming its functional activity in the mouse system. In addition, previous studies indicate that IL-39 (p19/EBI3) signals through gp130 based receptor complexes, and its functional activity has been reported in murine systems, with downstream effects converging on conserved chemotactic and inflammatory pathways 69, 70. As a limitation of our schematic (Figure 9), we moderated the annotation that “IL-39 is primarily secreted by B cells,” clarifying that this predominance is reported in murine contexts and that the precise cellular source in human sepsis remains to be defined 71. We used HL-60 and RAW264.7 cell lines as human neutrophil-like and murine macrophage-like models due to their well-characterized nature and widespread use in IL-12/IL-23 family signaling studies 72-75. While these cell lines may not fully capture the diversity of primary myeloid responses, they provide valuable insights into IL-39 signaling. Species-specific differences in macrophage and neutrophil responses to IL-39 are possible, as previous studies highlight transcriptional differences between human and murine cells 76-78. However, the high conservation of IL-39 subunits (IL-23p19 and EBI3) between humans and mice, with 72.45% and 60.43% amino acid identity, respectively, supports functional compatibility across species, particularly at the receptor-binding and signaling interfaces 79, 80. We also recognize that tissue origin influences immune cell responses, with macrophages from different tissues exhibiting distinct activation patterns 77, 81, 82. To address this, we repeated experiments with primary bone marrow-derived neutrophils and macrophages and found consistent results (Figures 7A and 8A). These findings suggest that while species- and tissue-specific factors may affect responses, the conserved nature of IL-39 ensures its relevance in both human and murine models. Finally, at the assayed time point, serum cytokines were unchanged whereas lung cytokine transcripts decreased after IL-39 neutralization; this pattern supports a predominantly local anti-inflammatory effect, and future work will quantify tissue proteins across multiple time points to link transcriptional and protein-level effects more definitively.

In summary, our study demonstrates that IL-39 may serve as a potential prognostic biomarker in clinical sepsis. In the present cohort, IL-39 demonstrated an AUC of 0.719 for predicting 90-day mortality, indicating moderate discriminatory ability, which is encouraging as an initial single-center observation. Given the limited sample size, larger, multicenter studies are warranted to determine whether the predictive performance of IL-39 can be further optimized and whether it adds value beyond established severity scores when used in combination. Neutralizing IL-39 antibody might alleviate symptoms and improve survival in sepsis, thereby laying a foundation for future research. Prospective, multi-center cohorts with protocolized capture of co-interventions (e.g., CRRT) and dose-finding studies that account for possible hormesis will be crucial next steps 39, 50, 83.

## Conclusions

The present study establishes IL-39 as a driver for sepsis progression. The elevated IL-39 levels in septic patients are associated with clinical severity and mortality, while preclinical models reveal that IL-39 exacerbates pulmonary injury and systemic inflammation by enhancing neutrophil infiltration through activation of the CXCL1-CXCR2 signaling pathway. These findings position IL-39 as a prognostic indicator and a potential therapeutic target for mitigating neutrophil-mediated tissue damage in sepsis.

## Supplementary Material

Supplementary materials and methods, figures and tables.

## Figures and Tables

**Table 1:** Clinical characteristics and biomarker levels in sepsis patients compared to healthy controls.

	Healthy controls (N = 56)	All sepsis patients (N = 108)	Sepsis survivors (N = 81)	Sepsis non-survivors (N = 27)	P value
Sex (M/F)	30/26	74/34	55/26	19/8	0.8109
Age, years	59 (18-71)	63 (18-82)	62 (18-82)	64 (26-79)	*0.4022*
WBC, 10^9/L	5.37 (4.46-7.83)	11.68 (0.78-51.84)	11.62 (0.78-35.91)	13.02 (2.17-51.84)	0.1458
CRP (ng/mL)	NA	165.5 (2-426)	138 (2-340)	205 (58-426)	0.0020
PCT (ng/mL)	NA	6.94 (0.05-277.49)	3.6 (0.05-143.99)	23.2 (0.76-277.49)	*0.0001*
SOFA	NA	5 (0-19)	5 (0-14)	9 (1-19)	*0.0003*
APACHE II	NA	13 (2-30)	12 (2-30)	18 (4-30)	*0.0046*
CRRT use, n (%)	NA	21 (19.4)	14 (17.3)	7 (25.9)	*0.4003*
Time to first RRT, days (CRRT users only)	NA	4.00 (3.00-6.50)	4.00 (3.00-6.00)	3.50 (2.25-11.50)	*0.9295*
RRT sessions, times (CRRT users only)	NA	6.00 (3.00-12.00)	4.00 (3.00-8.00)	9.00 (5.00-18.00)	*0.1242*

The analysis of survivors and non-survivors among sepsis patients was conducted using chi-square or Fisher's exact tests for categorical variables, and the Mann-Whitney U test for continuous variables.

**Table 2:** Infection focus, mechanical ventilation and pathogen identification at enrollment in sepsis patients.

Variable	Overall (N = 108)	Survivor (N = 81)	Non-survivor (N = 27)	P value
Infection focus: Pulmonary	47 (43.5%)	37 (45.7%)	10 (37.0%)	0.405
Infection focus: Abdominal	46 (42.6%)	31 (38.3%)	15 (55.6%)	
Infection focus: Urinary	9 (8.3%)	8 (9.9%)	1 (3.7%)	
Infection focus: Other/unknown	6 (5.6%)	5 (6.2%)	1 (3.7%)	
Mechanical ventilation: Yes	53 (49.1%)	42 (51.9%)	11 (40.7%)	0.377
Mechanical ventilation: No	55 (50.9%)	39 (48.1%)	16 (59.3%)	
Pathogen identified: Yes	5 (4.6%)	3 (3.7%)	2 (7.4%)	0.597
Pathogen identified: No/unknown	103 (95.4%)	78 (96.3%)	25 (92.6%)	

**Table 3:** AUC and optimal cutoff points with their corresponding validity indexes and predictive values for biomarkers and severity scores in relation to 90-day mortality.

Parameter	Cut off	AUC (95% CI)	Sensitivity (%)	Specificity (%)	Youden Index	*P* value
IL-39	79.70	0.719 (0.593-0.845)	59.3	85.2	0.444	*0.001*
SOFA	8.0	0.731 (0.605-0.857)	63.0	79.0	0.420	*< 0.001*
APACHE II	16.00	0.683 (0.562-0.803)	59.3	75.3	0.346	*0.003*
PCT	13.12	0.750 (0.647-0.854)	74.1	67.9	0.420	< 0.001
WBC	19.7	0.594 (0.461-0.727)	33.3	87.7	0.210	0.166

**Table 4:** Receiver operating characteristic (ROC) curves for sepsis-related severity scores and biomarkers in relation to 90-day mortality, along with their corresponding optimal cutoff points, validity indices, and predictive values.

Parameters	Standard error	Wald	95% CI	Odds ratio	*P* value
Univariate Cox models					
IL39	0.013	17.373	1.030 to 1.086	1.057	*<0.0001*
SOFA	0.054	19.741	1.144 to 1.415	1.272	*<0.0001*
APACHEII	0.029	9.277	1.032 to 1.157	1.093	*0.002*
PCT	0.002	17.224	1.005 to 1.015	1.010	*<0.0001*
WBC	0.020	4.886	1.005 to 1.088	1.046	*0.027*
Multivariate Cox models					
IL39	0.023	1.711	0.985 to 1.080	1.031	*0.191*
SOFA	0.084	1.989	0.955 to 1.326	1.125	*0.158*
APACHEII	0.045	0.087	0.903 to 1.079	0.987	0.768
PCT	0.003	1.677	0.998 to 1.011	1.004	0.195
WBC	0.023	0.124	0.964 to 1.054	1.008	0.725

## Abbreviations

ALI: Acute lung injury
APACHE II: Acute Physiology and Chronic Health Evaluation II
ARDS: Acute respiratory distress syndrome
AUC: Area under the receiver operating characteristic curves
CLP: Cecal ligation and puncture
ALT: Alanine aminotransferase
AST: Aspartate aminotransferase
COVID-19: Coronavirus Disease 2019
CRP: C-reactive protein
CXCL1: Chemokine (C-X-C motif) ligand 1
CXCR1: Chemokine (C-X-C motif) receptor 1
CXCR2: Chemokine (C-X-C motif) receptor 2
DMSO: Dimethylsulfoxide
EBi3: Epstein-Barr virus-induced gene 3
ELISA: Enzyme-linked immunosorbent assay
GVHD: Graft-versus-host disease
HE: Hematoxylin and eosin
ICU: Intensive care units
IHC: Immunohistochemistry
IL-1β: Interleukin-1 beta
IL-6: Interleukin-6
IL-6ST: IL-6 signal transducer
IL-12: Interleukin-12
IL-23: Interleukin-23
IL-23A: Interleukin-23 subunit alpha
IL-23p19: Interleukin-23 subunit p19
IL-23R: Interleukin-23 receptor
IL-27: Interleukin-27
IL-35: Interleukin-35
IL-39: Interleukin-39
MOF: Multiple organ failure
NETs: Neutrophil extracellular traps
PCT: Procalcitonin
rhIL-39: Recombinant human IL-39
RNA-seq: RNA sequencing
ROC: Receiver operating characteristic
RT-qPCR: Real-time quantitative polymerase chain reaction
SIRS: Systemic inflammatory response syndrome
SLE: Systemic lupus erythematosus
SOFA: Sequential Organ Failure Assessment
TNF-α: Tumor Necrosis Factor-alpha
TUNEL: Terminal deoxynucleotidyl transferase dUTP nick end labeling
WBC: White blood cell
