# Supplementary material for: Interleukin-39 aggravates sepsis by inducing self-sustaining neutrophil activation in the lungs via the CXCL1-CXCR2 pathway
Source: Int J Biol Sci. 2026 Jul 22;22(13):6945–68. doi: 10.7150/ijbs.124607 (PMC13540459; doi:10.7150/ijbs.124607)
Supplement: Supplementary file 1 [file ijbsv22p6945s1.pdf]

Supplementary Materials

Interleukin-39 aggravates sepsis by inducing self-sustaining neutrophil activation in the lungs via the CXCL1-CXCR2 pathway

Jinming Zhang[1, #], Jingting Liu[1, #], Yihui Wang[2, #], Yifan Xu[1, #], Robim M Rodrigues[3], Zihan Zhou[1], Jie Lu[1], Ziqiang Li[1], Xiaolin Wang[1], Jingjiao Li[1], Yong He[4], Fouad Lafdil [5,6,7,8], Ruidong Mo[1, *], Enqiang Mao[2, *], Qing Xie[1, *], Xiaogang Xiang[1, *]

[1] Department of Infectious Diseases, Translational Laboratory of Liver Diseases, Ruijin Hospital, Shanghai Jiao Tong University School of Medicine, Shanghai, 200025, China; [2] Department of Emergency, Ruijin Hospital, Shanghai Jiao Tong University School of Medicine, Shanghai, China; [3]Department of In Vitro Toxicology and Dermato-Cosmetology (IVTD), Faculty of Medicine and Pharmacy, Vrije Universiteit Brussel (VUB), Laarbeeklaan 103, B1090, Brussels, Belgium; [4]Shanghai Institute of Materia Medica (SIMM), Chinese Academy of Sciences, Shanghai, 201203, China. [5] Université Paris-Est Créteil (UPEC), Créteil, France; [6]INSERM, U938, Centre de Recherche Saint-Antoine (CRSA), Paris, France; [7]Sorbonne University, Paris, France. [8]Institut Universitaire de France (IUF), Paris, France.

# The first three authors contributed equally.

*** Correspondence:**

Xiaogang Xiang, Department of Infectious Diseases, Translational Laboratory of Liver Diseases, Ruijin Hospital, Shanghai Jiao Tong University School of Medicine, Shanghai, 200025, China. Email: xiaogang.xiang@shsmu.edu.cn or shine-xxg@163.com

Qing Xie, Department of Infectious Diseases, Translational Laboratory of Liver Diseases, Ruijin Hospital, Shanghai Jiao Tong University School of Medicine, Shanghai, 200025, China. Email: xieqingrjh@163.com

Enqiang Mao, Department of Emergency, Ruijin Hospital, Shanghai Jiao Tong University School of Medicine, Shanghai, China. Email: maoeq@yeah.net

Tables of contents

**Supplementary Material and methods**		**Page**
**Patients and sample preparation**	••••••••••••••••••	**4**
**Mice**	••••••••••••••••••	**4**
**CLP model**	••••••••••••••••••	**4**
**Enzyme-linked immunosorbent assay (ELISA)**	••••••••••••••••••	**6**
**Cell culture**	••••••••••••••••••	**6**
**Supplementary References**	••••••••••••••••••	**7**
**Supplementary Tables S1-S11**	••••••••••••••••••	**8**
**Supplementary Figures S1-S10**	••••••••••••••••••	**19**

Supplementary Material and methods

Patients and sample preparation

Serum samples from septic patients were obtained from Department of Emergency, Shanghai Ruijin Hospital, affiliated to Shanghai Jiao Tong University School of Medicine. The experiment protocol was approved by *Human Ethics Committee of Ruijin Hospital* (Ethics committee reference number:2020-353), and written informed consents were obtained from the subjects. Inclusion criteria were as follows: (i) age ≥ 18 years; (ii) diagnosis of sepsis defined as suspected infection plus organ dysfunction (total SOFA score ≥ 2); (iii) enrollment within 72 hours after the diagnosis of sepsis; (iv) non-pregnant throughout the study period [1].

Mice

Male C57BL/6J mice (6 to 8 weeks old) were purchased from Beijing Vital River Laboratory Animal Technology Co. Ltd. All the experimental animals were housed in cages (with a maximum of five mice per cage) and maintained in a temperature (24±1℃) and light controlled facility (12 h:12 h light-dark cycle). Animals were randomly assigned to groups and outcome assessors were blinded to treatment.

CLP model

Polymicrobial sepsis in mice was induced by cecal ligation and puncture (CLP) [2]. Briefly, mice were anesthetized by intraperitoneal injection of 1% pentobarbital. The cecum of the anesthetized mice was exposed after a midline laparotomy. The distal portion of exposed cecum (1cm) was ligated with 3-0 silk suture and two punctures were made using a 20G needle for one pass. Subsequently, the cecum was replaced into the abdomen cavity, and the incision was sutured with 4-0 suture. Sham

operated mice underwent the same procedures except for cecal puncture. Cecal ligation and puncture (CLP) was performed according to established guidelines. Model severity was controlled by adjusting ligation length, needle gauge, and puncture number. Based on pilot optimization and prior reports, we used a single cecal puncture with a 20-gauge needle and gently expressed a small amount of fecal material. Under our conditions, this protocol reproducibly generated an acute severe sepsis phenotype in which most fatal events occurred between 18h and 48h post-CLP, rather than immediate (<12 h) or very delayed (>72 h) lethality, providing a clinically relevant early intervention window for assessing IL-39-mediated immunomodulation [3-7]. The ligation length was standardized relative to the ileocecal valve to minimize variability [3, 4] (**Figure S6**).

Rectal temperature was monitored in all mice at hourly intervals from immediately after CLP induction (0 h) to 8 hours post-surgery. Peripheral blood was collected from mice at 0, 6, and 8 hours after CLP induction. Serum was then isolated and analyzed for the liver injury indicators alanine aminotransferase (ALT) and aspartate aminotransferase (AST). Serum samples were collected from mice at 0 h and 6 h post-CLP, and levels of IFN-γ and IL-6 were measured by ELISA. Heart, liver, lung, and kidney tissues were collected from mice at 0 h, 6 h, and 8 h post-CLP, subjected to H&E staining, and then evaluated for organ injury scoring.

Two hours after CLP surgery, mice were intraperitoneally administered with either recombinant human IL-39 (rhIL-39; 9990-IL-050, R&D Systems, USA), dissolved in PBS, or an anti-IL-39 antibody (DETAIBIO, China). For antibody-dose exploration, anti-IL-39 was tested at 4, 5 or 8 mg/kg; with 5 mg/kg conferring the clearest benefit (**Figure 4, Figure S2**).

In exploratory arms, we assessed two antibodies targeting the IL-39 subunits as comparators to a heterodimer-specific IL-39 neutralizing antibody (**Figure S2**). These included anti-IL-23p19 (HY-P990217, MedChemExpress, USA), administered at 2 mg/kg and 4 mg/kg, and anti-EBI3 (MABF848,

Merck Millipore, USA), used at a single dose of 2 mg/kg. All treatments were given 2 hours post-CLP.

To further determine whether CXCR2 signaling contributed to the biological effects of IL-39, additional CLP mice were treated with IL-39 in the presence or absence of the CXCR2 inhibitor SB 265610 (Selleck Chemicals, Houston, TX, USA) two hours after CLP surgery. Notably, treatment with the CXCR2 inhibitor at 2 mg/kg did not improve survival in the IL-39-exacerbated CLP model (**Figure S5**). In contrast, a lower dose of 0.5 mg/kg significantly reduced mortality in the same model (**Figure 8F**), suggesting that CXCR2 inhibition may need to be maintained within an appropriate therapeutic range.

Enzyme-linked immunosorbent assay (ELISA)

Whole blood from mice was collected, and the serum was separated by centrifugation, and stored at -80 °C. Serum and tissue IL-6, IL-1β, and TNF-α levels were measured using the Mouse IL-6 One-Step ELISA kit (EK206EGA, MultiSciences, China), the Mouse IL-1β One-Step ELISA kit (EK201BEG, MultiSciences, China), and the Mouse TNF-α One-Step ELISA kit (EK282EG, MultiSciences, China), respectively. All samples were measured in duplicate, and the results were expressed as pg/mL. All samples were measured in duplicate.

Cell culture

Bone marrow cells were harvested from mice and cultured in complete RPMI 1640 medium containing M-CSF (20 ng/mL) for 5 days to induce differentiation into bone marrow derived macrophages (BMDMs). Subsequently, these macrophages were stimulated with rhIL-39 for 0 or 3 hours. Cells were then collected, and mRNA was extracted to analyze the expression levels of

downstream inflammatory cytokines. RAW264.7 cells were stimulated with rhIL-39 for 0 and 3 hours, after which cells were harvested for mRNA extraction to assess downstream inflammatory factor expression.

Supplementary References

1. Singer M, Deutschman CS, Seymour CW, Shankar-Hari M, Annane D, Bauer M, et al. The Third International Consensus Definitions for Sepsis and Septic Shock (Sepsis-3). Jama. 2016; 315: 801-10.
2. Rittirsch D, Huber-Lang MS, Flierl MA, Ward PA. Immunodesign of experimental sepsis by cecal ligation and puncture. Nature Protocols. 2008; 4: 31-6.
3. Rittirsch D, Huber-Lang MS, Flierl MA, Ward PA. Immunodesign of experimental sepsis by cecal ligation and puncture. Nat Protoc. 2009; 4: 31-6.
4. Drechsler S, Osuchowski M. Cecal Ligation and Puncture. Methods Mol Biol. 2021; 2321: 1-8.
5. Ruiz S, Vardon-Bounes F, Merlet-Dupuy V, Conil JM, Buléon M, Fourcade O, et al. Sepsis modeling in mice: ligation length is a major severity factor in cecal ligation and puncture. Intensive Care Med Exp. 2016; 4: 22.
6. Iskander KN, Craciun FL, Stepien DM, Duffy ER, Kim J, Moitra R, et al. Cecal ligation and puncture-induced murine sepsis does not cause lung injury. Crit Care Med. 2013; 41: 159-70.
7. Torrente Y, El Fahime E, Caron NJ, Del Bo R, Belicchi M, Pisati F, et al. Tumor necrosis factor-alpha (TNF-alpha) stimulates chemotactic response in mouse myogenic cells. Cell Transplant. 2003; 12: 91-100.

Supplementary Tables:

Table S1. Baseline IL-39, total SOFA score, and SOFA organ subscores in survivors *vs.* non-survivors for 90-day mortality.

Variable at enrollment	Survivors (n=81)	Non-survivors (n=27)	P value
IL-39 (pg/mL)	66.46 [60.49–74.73]	83.54 [65.65–94.22]	*0.00070*
Total SOFA score	5.00 [3.00–7.00]	9.00 [5.00–11.00]	*0.00032*
Respiratory SOFA subscore	2.00 [1.00–2.00]	2.00 [2.00–2.00]	*0.2951*
Coagulation SOFA subscore	0.00 [0.00–2.00]	1.00 [0.50–2.00]	0.1206
Liver SOFA subscore	1.00 [0.00–1.00]	2.00 [1.00–3.00]	*<0.0001*
Cardiovascular SOFA subscore	0.00 [0.00–1.00]	1.00 [0.00–3.00]	*<0.0001*
Central nervous system SOFA subscore	1.00 [0.00–1.00]	1.00 [0.50–2.00]	*0.0284*
Renal SOFA subscore	0.00 [0.00–1.00]	0.00 [0.00–0.50]	*0.0994*
Age (years)	62.00 [43.00–71.00]	64.00 [57.00–69.00]	*0.4022*
APACHE II score	12.00 [9.00–15.00]	18.00 [13.00–20.00]	*0.0046*
CRRT use, n (%)	14 (17.3)	7 (25.9)	*0.4003*
Time to first CRRT (days, CRRT users only)	4.00 [3.00–6.00]	3.50 [2.25–11.50]	*0.9295*
CRRT sessions (times, CRRT users only)	4.00 [3.00–8.00]	9.00 [5.00–18.00]	*0.1242*

Data are presented as median [IQR] or n (%), as appropriate. P values calculated by Mann–Whitney U test for continuous variables and chi-square or Fisher's exact tests for categorical variables. SOFA subscores: respiratory, coagulation, liver, cardiovascular, central nervous system, renal. Survivors: n =81; Non-survivors: n=27.

Table S2. Multivariable logistic regression analyses for 90-day mortality.

Model	Predictor	Adjusted OR (per unit as specified)	95% CI	P value
Model 1: IL-39 only	IL-39 (per 10 pg/mL)	1.89	1.35 – 2.66	0.0002
Model 2: IL-39 + SOFA organ subscores	IL-39 (per 10 pg/mL)	1.41	0.91 – 2.19	0.13
	Liver SOFA subscore	ns	ns	ns
	Respiratory SOFA subscore	ns	ns	ns
	Coagulation SOFA subscore	ns	ns	ns
	Cardiovascular SOFA subscore	ns	ns	ns
	CNS SOFA subscore	ns	ns	ns
	Renal SOFA subscore	ns	ns	ns
Model 3: Model 2 + age, sex, APACHE II	IL-39 (per 10 pg/mL)	1.31	0.76 – 2.27	0.34
	Liver SOFA subscore	ns	ns	ns
	Age (per year)	ns	ns	ns
	Sex	ns	ns	ns
	APACHE II score	ns	ns	ns

OR: odds ratio; CI: confidence interval; ns: not significant.

Table S3. Multivariable logistic regression analyses for 90-day mortality after additionally adjusting for infection focus and mechanical ventilation.

Model	Predictor	Adjusted OR (per unit as specified)	95% CI	P value
Model: IL-39 + infection focus + mechanical ventilation	IL-39 (per 10 pg/mL)	2.19	1.50 – 3.20	<0.0001
	Infection focus: Abdominal vs Pulmonary	ns	ns	ns
	Infection focus: Urinary vs Pulmonary	ns	ns	ns
	Infection focus: Other/unknown vs Pulmonary	ns	ns	ns
	Mechanical ventilation: Yes vs No	ns	ns	ns

Table S4. Sensitivity analyses evaluating the impact of limited microbiological information on the association between IL-39 and 90-day mortality.

Analysis	N	IL-39 Adjusted OR (per 10 pg/mL)	95% CI	P value
Primary analysis (full cohort)	108	2.19	1.50 – 3.20	<0.0001
Sensitivity 1: excluding patients with pathogen identified	103	2.09	1.44 – 3.05	0.0001
Sensitivity 2: additionally adjusting for pathogen identified	108	2.21	1.51 – 3.23	<0.0001

Additional note: In Sensitivity 2, pathogen identified (Yes vs No/unknown) had OR 2.32 (95% CI 0.32 – 17.04), P=0.41 (imprecise due to small n).

Table S5. Clinical characteristics and biomarker levels in sepsis patients compared to healthy controls for 28-day mortality.

	Healthy controls (N=56)	All sepsis patients (N = 108)	Sepsis survivors (N = 86)	Sepsis non-survivors (N = 22)	P value
**Sex (M/F)**	30/26	74/34	57/29	17/5	0.4632
**Age, years**	59 (18-71)	62.50 (18.00-82.00)	62.00 (18.00-82.00)	63.50 (26.00-79.00)	0.5645
**WBC, 10^9/L**	5.37 (4.46-7.83)	11.68 (0.78-51.84)	11.65 (0.78-35.91)	12.59 (2.17-51.84)	0.4501
**CRP (ng/mL)**	NA	165.50 (2.00-426.00)	138.00 (2.00-340.00)	207.50(106.00-426.00)	0.0029
**PCT (ng/mL)**	NA	6.94 (0.05-277.49)	4.37 (0.05-196.70)	41.05 (1.35-277.49)	<*0.0001*
**SOFA**	NA	5.00 (0.00-19.00)	5.00 (0.00-14.00)	9.00 (1.00-19.00)	*0.0001*
**APACHE II**	NA	13.00 (2.00-30.00)	13.00 (2.00-30.00)	17.00 (4.00-30.00)	*0.0453*
**RRT use, n (%)**	NA	21 (19.4)	14 (16.3)	7 (31.8)	*0.1306*
**Time to first RRT, days (CRRT users only)**	NA	4.00 (3.00-6.50)	4.00 (3.00-6.00)	3.50 (2.25-11.50)	*0.9295*
**RRT sessions, times (CRRT users only)**	NA	6.00 (3.00-12.00)	4.00 (3.00-8.00)	9.00 (5.00-18.00)	*0.1242*

The analysis of survivors and non-survivors among sepsis patients was conducted using chi-square or Fisher's exact tests for categorical variables, and the Mann-Whitney U test for continuous variables.

Table S6. AUC and optimal cutoff points with their corresponding validity indexes and predictive values for biomarkers and severity scores in relation to 28-day mortality

Parameter	Cut off	AUC (95% CI)	Sensitivity (%)	Specificity (%)	Youden Index	*P* value
IL-39	75.32	0.751 (0.625-0.876)	72.7	76.7	0.495	*0.0001*
SOFA	8.00	0.764 (0.640-0.888)	68.2	77.9	0.461	0.0001
APACHE II	16.00	0.639 (0.502-0.775)	59.1	73.3	0.323	*0.046*
PCT	13.12	0.774 (0.652-0.896)	77.3	66.3	0.436	*0.0001*
WBC	16.43	0.553 (0.415-0.690)	36.4	77.9	0.143	0.454

Table S7. Receiver operating characteristic (ROC) curves for sepsis-related severity scores and biomarkers in relation to 28-day mortality, along with their corresponding optimal cutoff points, validity indices, and predictive values.

Parameters	Standard error	Wald	95% CI	Odds ratio	*P* value
Univariate Cox models					
IL39	0.015	17.497	1.034 to 1.097	1.065	*<0.0001*
SOFA	0.058	19.459	1.154 to 1.451	1.294	*<0.0001*
APACHEII	0.032	5.124	1.010 to 1.146	1.076	*0.024*
PCT	0.003	14.016	1.005 to 1.015	1.010	*0.0002*
WBC	0.023	2.623	0.992 to 1.085	1.038	0.105
Multivariate Cox models					
IL39	0.026	4.573	1.005 to 1.112	1.057	*0.032*
SOFA	0.091	2.187	0.957 to 1.368	1.144	0.139
APACHEII	0.052	2.015	0.839 to 1.028	0.929	0.156
PCT	0.004	1.286	0.997 to 1.011	1.004	0.257
WBC	0.026	0.003	0.952 to 1.054	1.001	0.958

Table S8. The 28-day mortality according to IL-39 group (high vs low) defined by the ROC-derived cutoff (75.32 pg/mL).

Comparison	Group A (n)	Group B (n)	28-day mortality, n/N (%)	P value
IL-39 group (cutoff 75.32 pg/mL)	Low (n=72)	High (n=36)	6/72 (8.3%) vs 16/36 (44.4%)	<0.0001
CRRT use	No (n=87)	Yes (n=21)	15/87 (17.2%) vs 7/21 (33.3%)	0.1306

Data are presented as median [IQR] or n (%), as appropriate. P values were calculated using the Mann–Whitney U test for continuous variables and the chi-square test or Fisher's exact test for categorical variables, as appropriate. IL-39 high vs low was defined using the ROC-derived cutoff of 75.32 pg/mL.

Table S9. Multivariable logistic regression analysis of 28-day mortality incorporating IL-39 group and CRRT use.

Outcome	Predictor	Adjusted OR	95% CI	P value
28-day mortality	IL-39 High vs Low	8.97	3.04 – 26.47	<0.0001
28-day mortality	CRRT use (Yes vs No)	2.54	0.76 – 8.50	0.1299

Multivariable logistic regression was performed to estimate odds ratios (ORs) with 95% confidence intervals (CIs) for 28-day mortality. IL-39 group was defined using the ROC-derived cutoff (75.32 pg/mL), and CRRT use (yes/no) was defined based on the leftmost column recording dialysis initiation day and session counts.

Table S10. Mouse primers used in RT-qPCR analysis

Gene	Forward primers (5'-3')	Reverse primers (5'-3')
18s	GCAATTATTCCCCATGAACG	GGCCTCACTAAACCATCCAA
Il23p19	ATGCTGGATTGCAGAGCAGTAA	AGCTGTTGGCACTAAGGGCTCAGT
Ebi3	GGTACCGCTCTCGTGGCTCTAAGCC	GGATCCGGGCTTATGGGGTGCACTT
Il6st	TTACTACGTGAATGCCAGCTACA	GACGTGGTTCTGTTGATGACA
Il23r	AACAACAGCTCGGATTTGGTAT	ATGACCAGGACATTCAGCAGT
Il4	GGTCTCAACCCCCAGCTAGT	GCCGATGATCTCTCTCAAGTGAT
Il1b	TTCAGGCAGGCAGTATCACTC	GAAGGTCCACGGGAAAGACAC
Tnfa	CAGGCGGTGCCTATGTCTC	CGATCACCCCGAAGTTCAGTAG
Ifny	TCAGGTAGTAACAGGCTGTCC	CATTCGGGTGTAGTCACAGTT
Il17a	TCAGCGTGTCCAAACACTGAG	TCAGCGTGTCCAAACACTGAG
Il17f	TGCTACTGTTGATGTTGGGAC	CAGAAATGCCCTGGTTTTGGT
Foxp3	CACCTATGCCACCCTTATCCG	CATGCGAGTAAACCAATGGTAGA
Cxcl1	ACTGCACCCAAACCGAAGTC	TGGGGACACCTTTTAGCATCTT

Table S11. Human primers used in RT-qPCR analysis

Gene	Forward primers (5’-3’)	Reverse primers (5’-3’)
Gapdh	GGAGCGAGATCCCTCCAAAAT	GGCTGTTGTCATACTTCTCATGG
Cxcr1	CTGACCCAGAAGCGTCACTTG	CCAGGACCTCATAGCAAACTG
Cxcr2	CCTGTCTTACTTTTCCGAAGGAC	TTGCTGTATTGTTGCCCATGT
Cxcl1	CCAGACCCGCCTGCTGAG	CCACGGACGCTCCTGCTG

Supplementary Figures

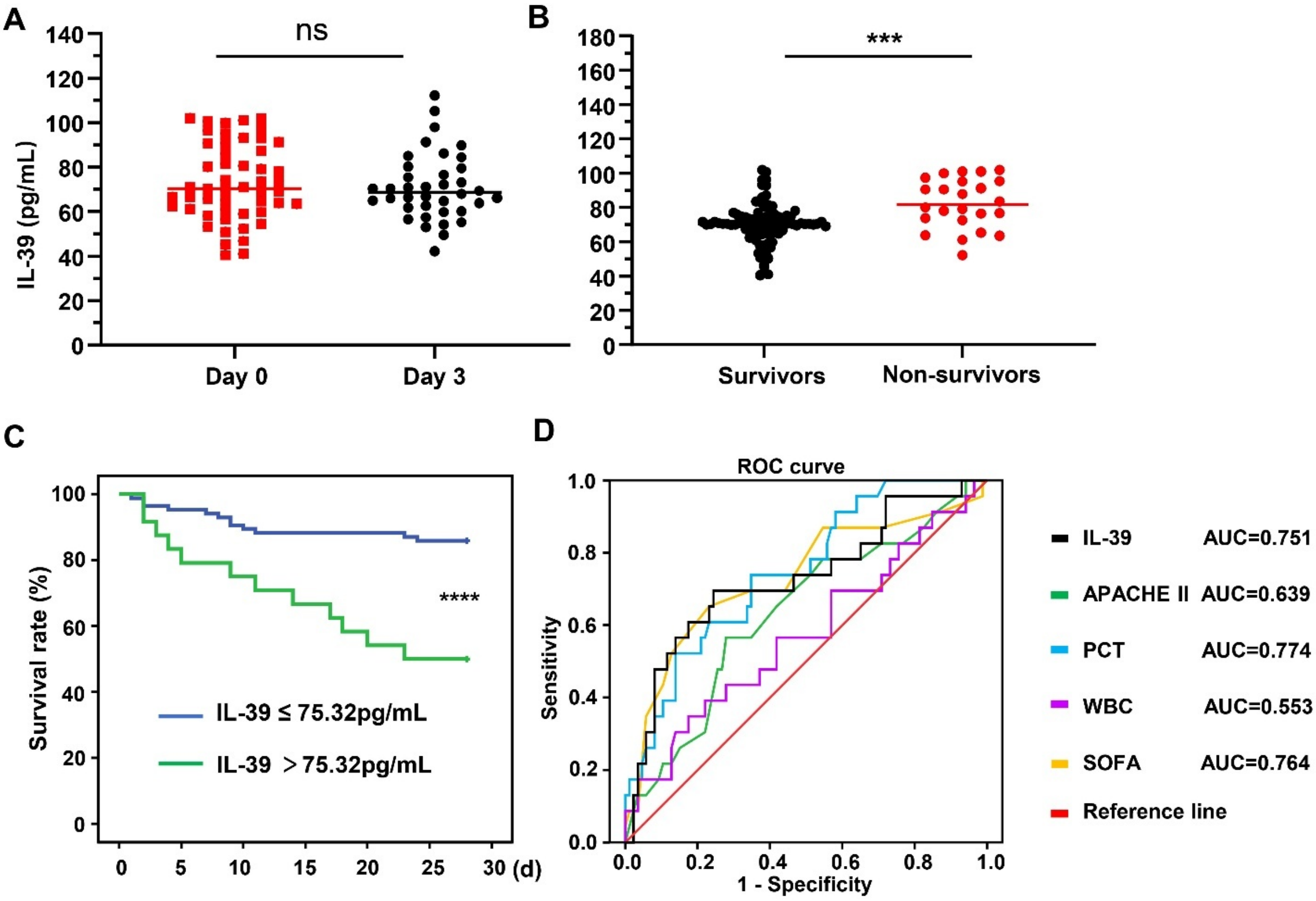

Figure S1. Associations between serum IL-39 levels and clinical outcomes in sepsis patients (**A**) Serum IL-39 levels measured by ELISA at hospital admission day 0 and on day 3. Data are presented as median with interquartile range. P value was determined by Mann–Whitney U test. **(B)** The levels of IL-39 in sepsis survivors (n= 86) and non-survivors (n= 22) upon admission with 28-day mortality as the endpoint. Data are presented as median with interquartile range. P value was determined by Mann–Whitney U test. (**C**) Kaplan–Meier curves by IL-39 cutoff with 28-day mortality as the endpoint. (**D**) ROC curves for predicting 28-day mortality in septic patients using IL-39, SOFA, APACHE II, PCT, and WBC levels at admission.

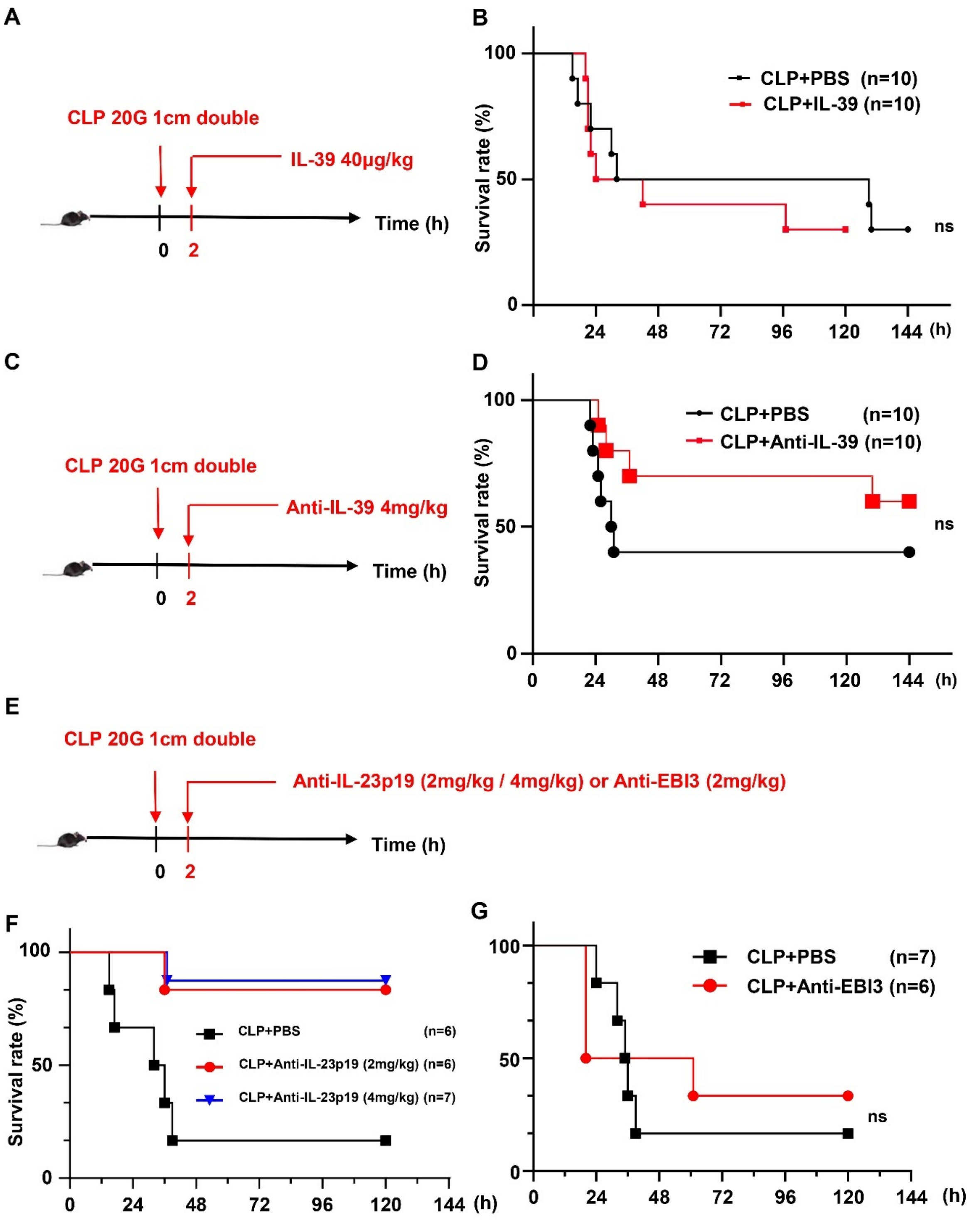

Figure S2. Survival of CLP mice treated with rhIL-39, anti-IL-23p19 or anti-EBi3. (**A-B**) Survival rates for CLP mice treated with 40 μg/kg recombinant human IL-39 (rhIL-39) (n=10 per group). **(C-D)** Survival rates of CLP-induced septic mice following IL-39 neutralizing antibody (Anti-IL-39) intervention (n=10 per group). (**E-G**) Survival rates of CLP-induced septic mice following IL-23A or EBi3 neutralizing antibodies (Anti-IL-23p19 or Anti-EBI3) intervention.

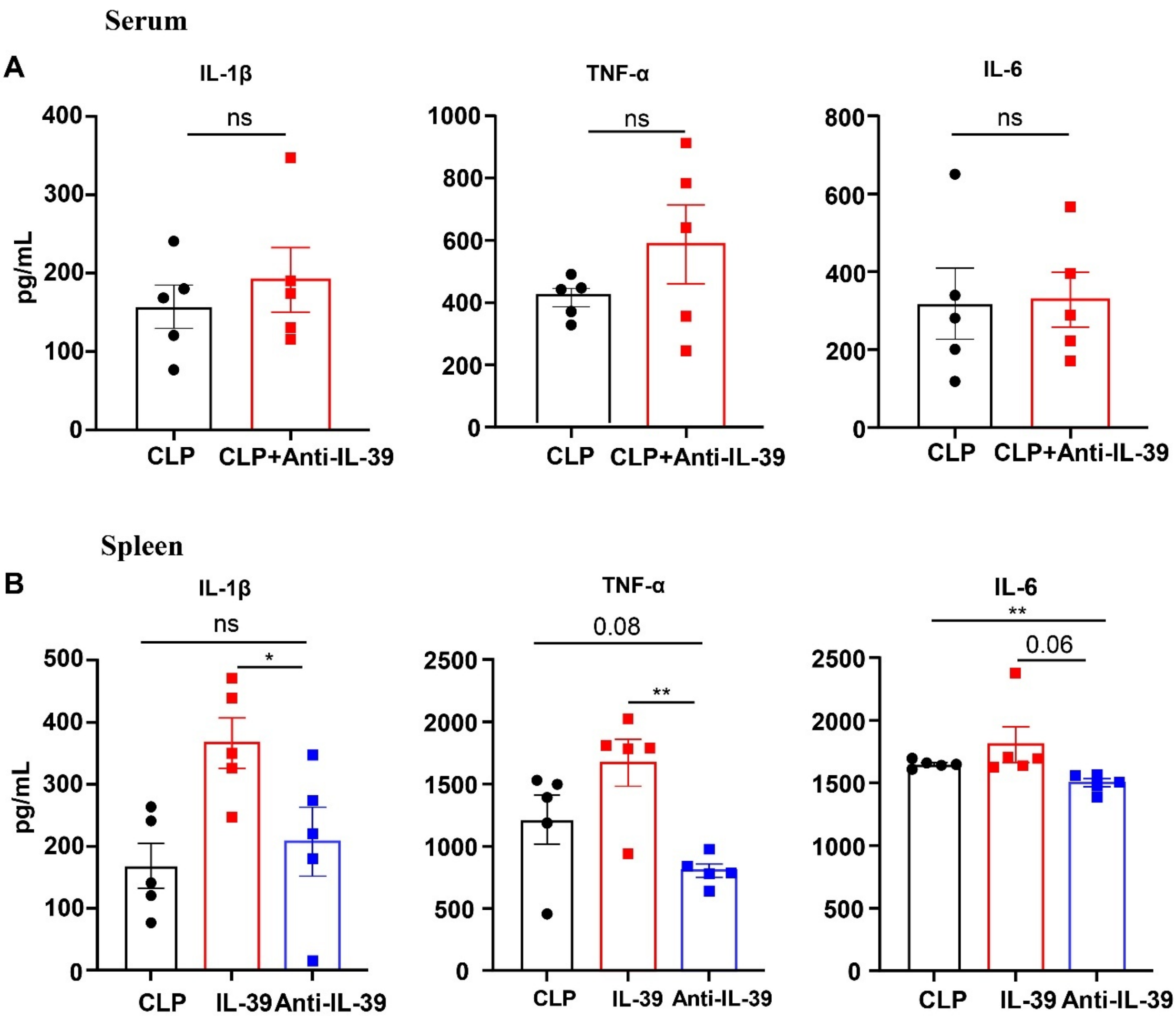

Figure S3. Anti-IL-39 neutralization shows no detectable changes in serum cytokines but partially reduces spleen inflammatory cytokine levels 14 h after intervention. (**A**) ELISA measurement of serum inflammation-related cytokine levels (IL-1β, TNF-α and IL-6) in mice 14 hours after anti-IL-39 intervention (n= 5 per group). (**B**) ELISA measurement of spleen tissue inflammation-related cytokine levels (IL-1β, TNF-α and IL-6) in mice 14 hours after anti-IL-39 intervention (n= 5 per group). The bars represent mean ± SEM.

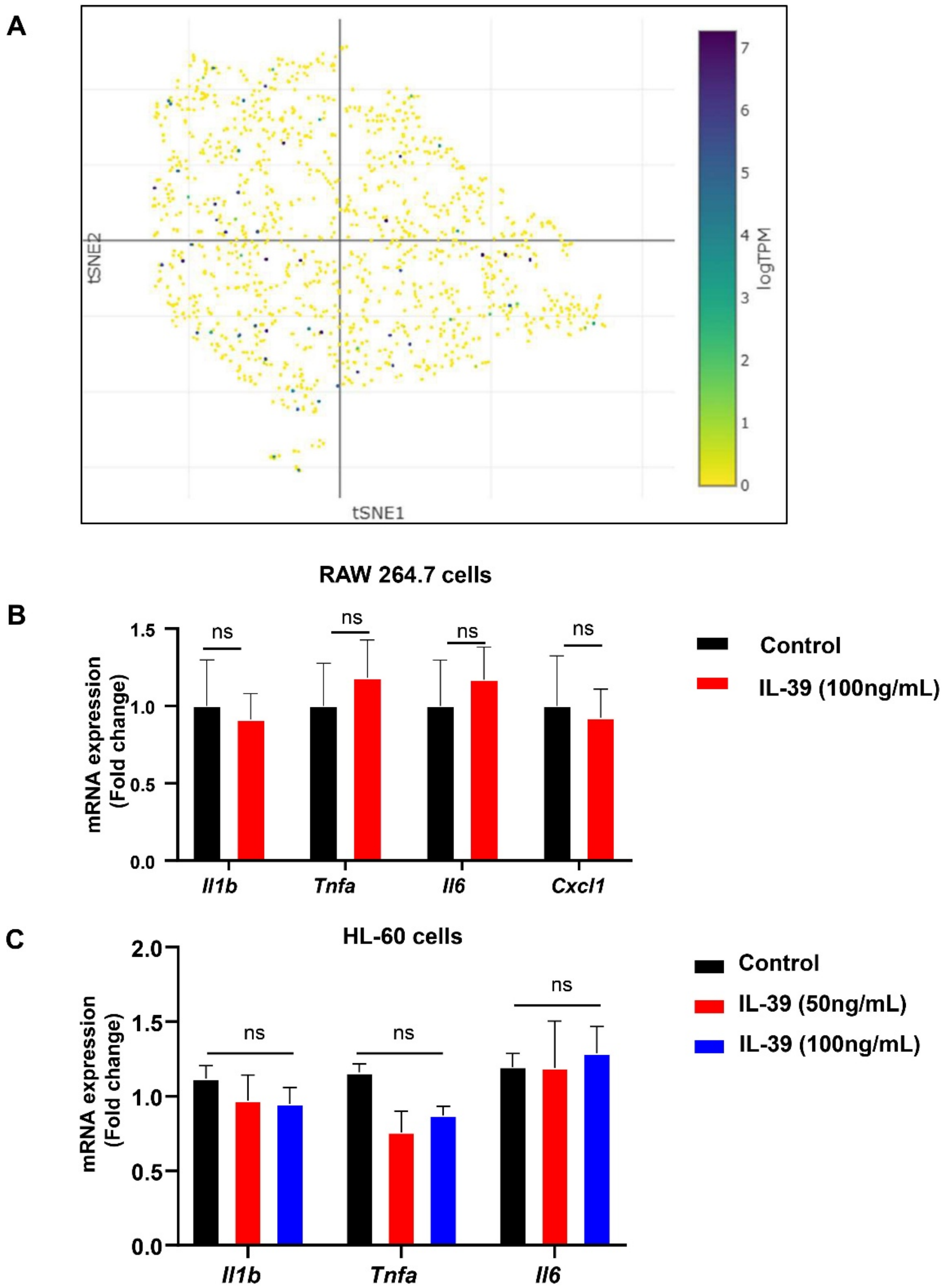

Figure S4. IL-39 Activates Pro-Inflammatory Signaling Across Human and Murine Systems (**A**) IL-6ST receptor expression on pulmonary neutrophils in septic patients, analyzed using publicly available single-cell datasets from the Single Cell Portal. **(B)** Real-time PCR analysis of inflammation-related genes in RAW 264.7 cells treated with recombinant human IL-39 (rhIL-39) for 48 hours. (**C**) Real-time PCR analysis of inflammation-related genes in HL-60 cells treated with recombinant human IL-39 (rhIL-39) for 48 hours. The bars represent mean ± SEM.

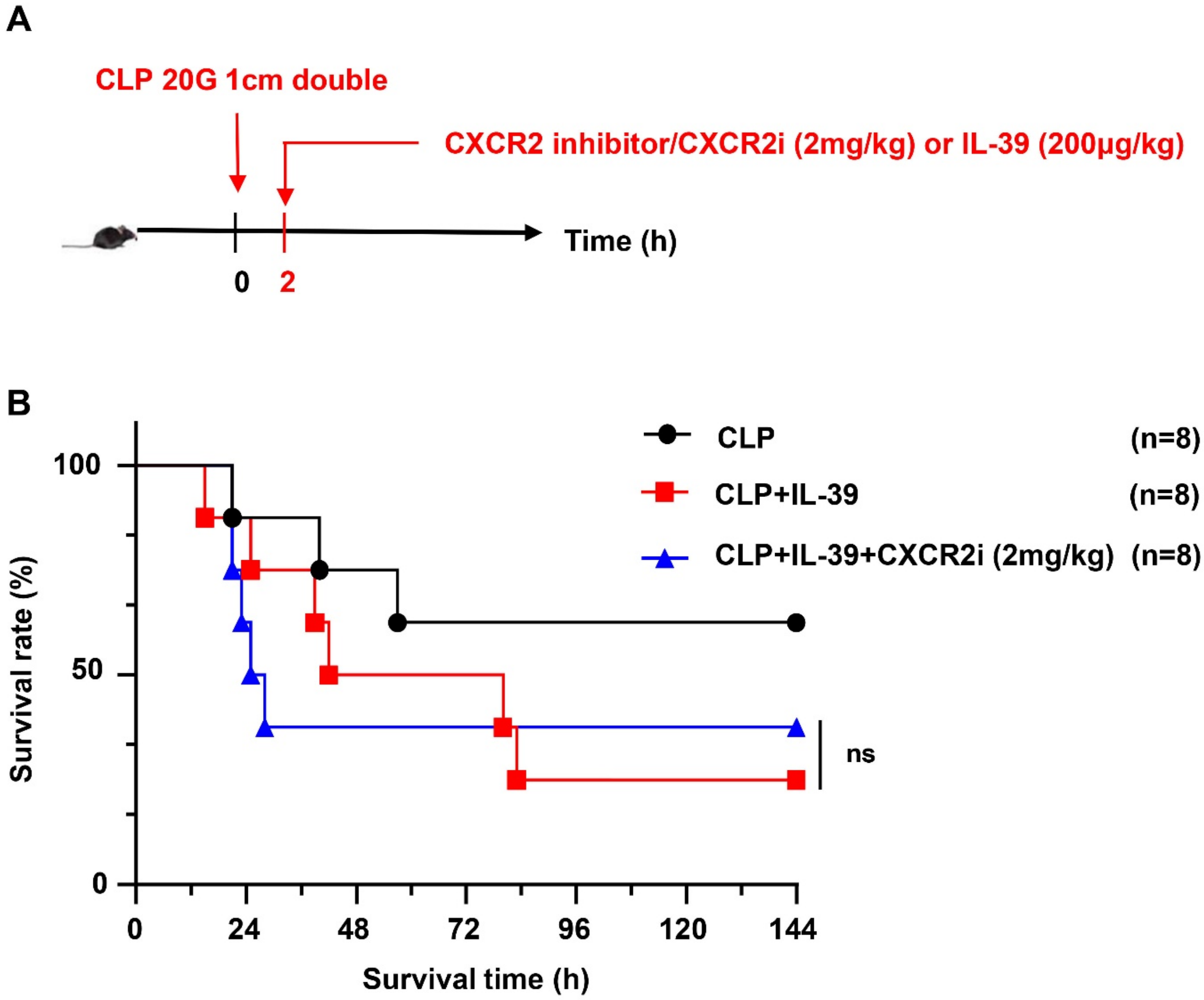

Figure S5. High dose of CXCR2 inhibitor cannot reduce the mortality in sepsis. (**A**) A schematic timeline of the experimental procedure for the survival rates of CLP mice treated with CXCR2 inhibitor (CXCR2i, 2mg/kg) or IL-39 (200mg/kg). (**B**) Survival rates for CLP mice treated with 2mg/kg CXCR2i (n=8 per group).

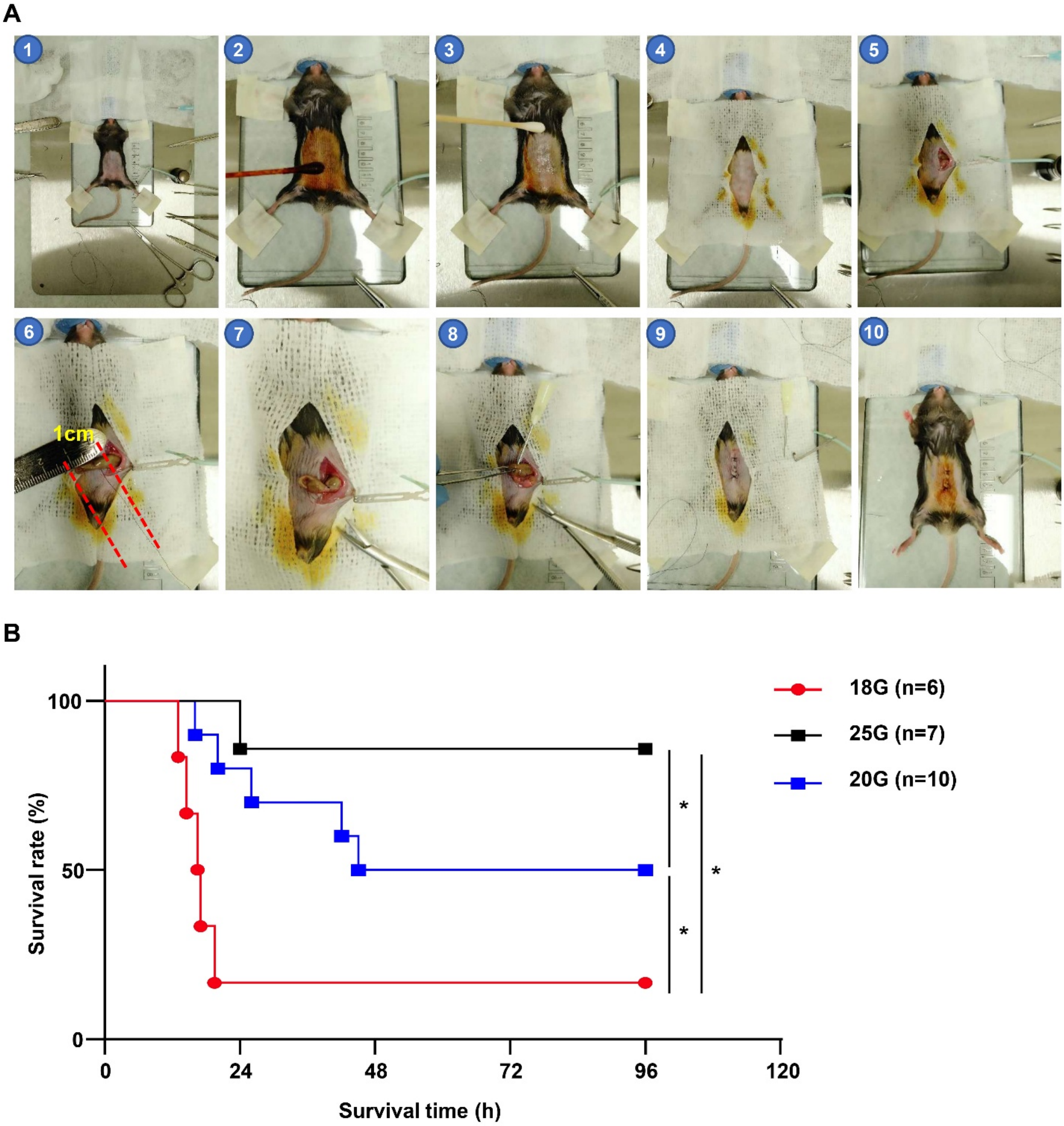

Figure S6. Establishment of the CLP Sepsis Model and the Impact of Needle Gauge on Post-CLP Survival. (A) The CLP (cecal ligation and puncture) procedure in mice. **(B)** Survival curves of mice subjected to cecal ligation and puncture (CLP) using different needle gauges (18G, 20G, 25G).

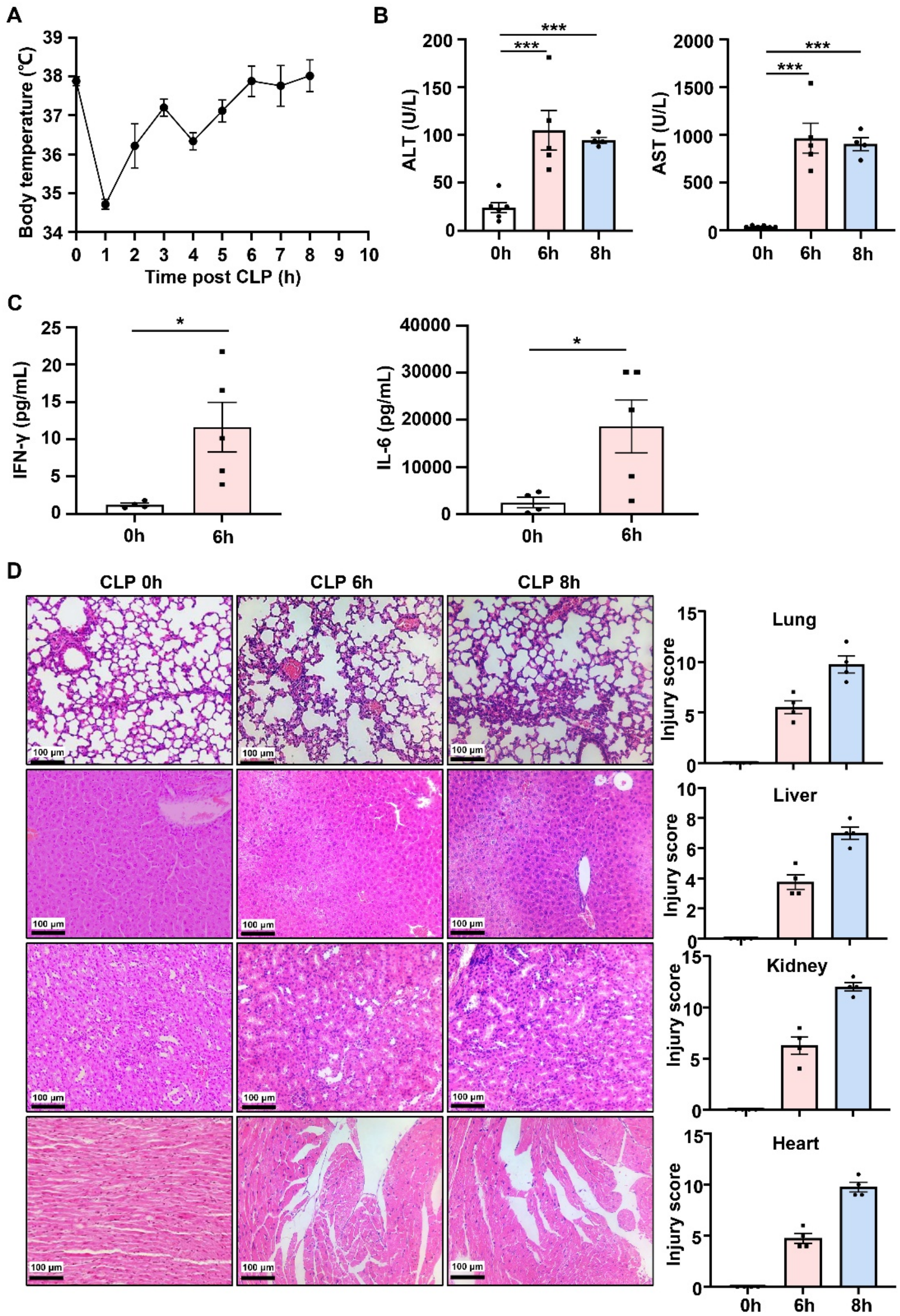

Figure S7. Time-Dependent Physiological, Biochemical, and Histopathological Changes in CLP-Induced Sepsis. (A) Body temperature of mice monitored at the indicated time points after cecal ligation and puncture (CLP)**. (B)** Serum ALT and AST levels measured at 0, 6, and 8 h post-CLP. **(C)** Serum IFN-γ and IL-6 levels measured at the indicated time points post-CLP. **(D)** Representative H&E staining of lung, liver, kidney, and heart tissues collected at 0, 6, and 8 h post-CLP, with corresponding organ injury scores shown on the right. The bars represent mean ± SEM.

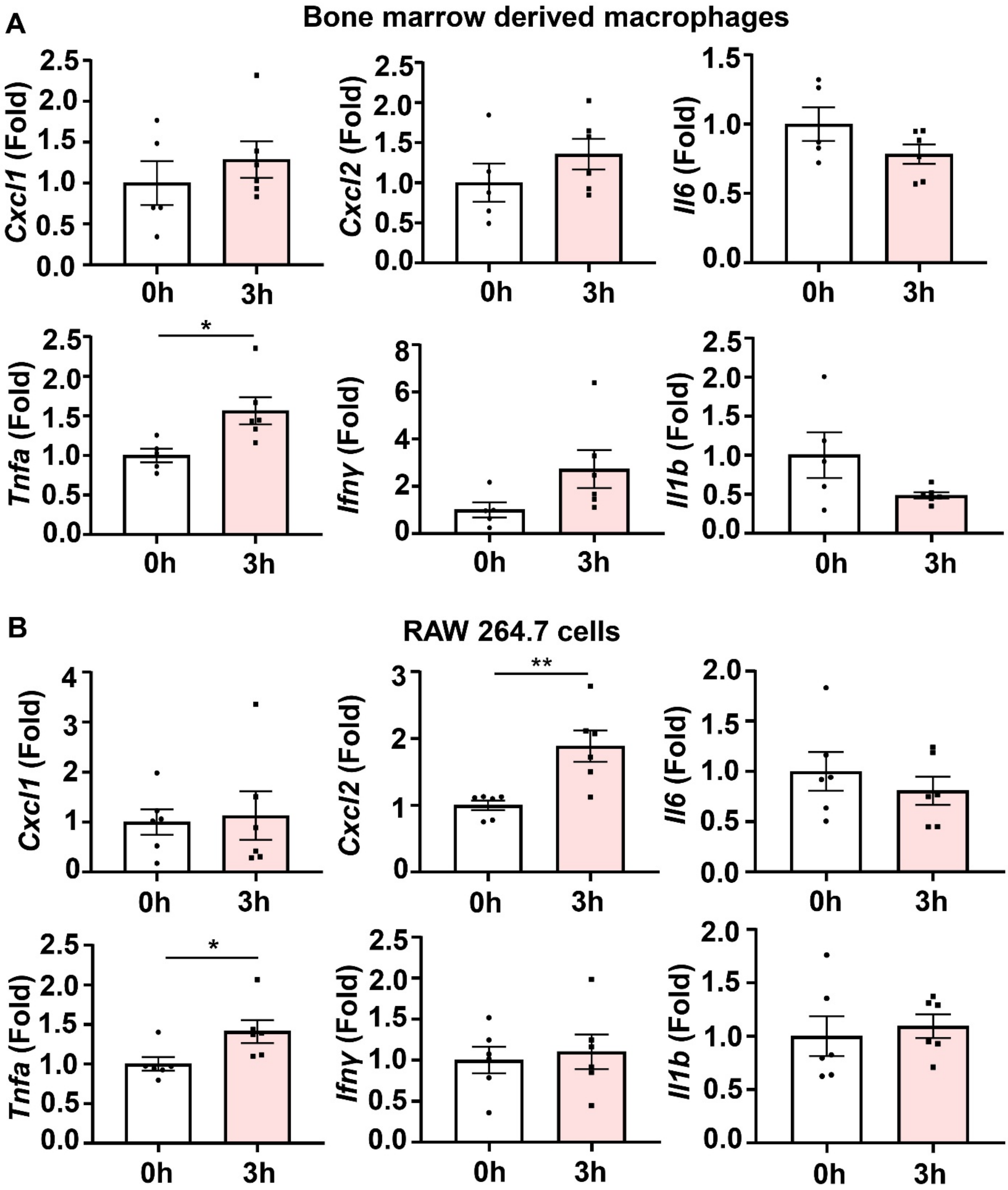

Figure S8. IL-39 Induces Chemokine and Cytokine Gene Expression in Macrophages. (A) RT-qPCR analysis of *Cxcl1*, *Cxcl2*, *Il6*, *Tnfa*, *Ifng*, and *Il1b* in bone marrow–derived macrophages at 0 h and 3 h after IL-39 stimulation. **(B)** RT-qPCR analysis of the indicated genes in RAW 264.7 cells at 0 h and 3 h following IL-39 stimulation. The bars represent mean ± SEM.

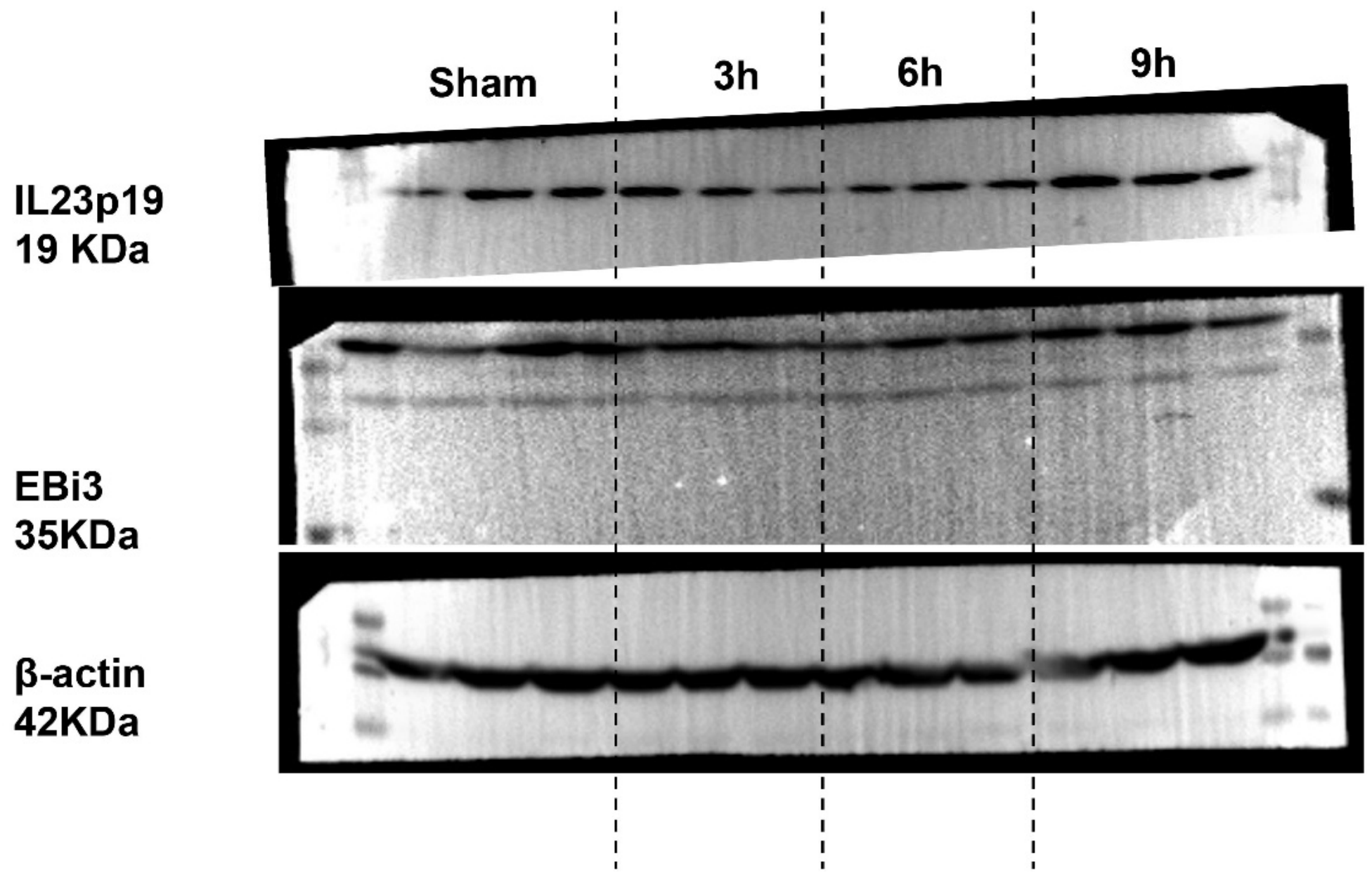

Figure S9. Unprocessed, full-length blots corresponding to the western blot panels (IL-23p19, EBi3, β-actin). Raw images for Figure 2D.

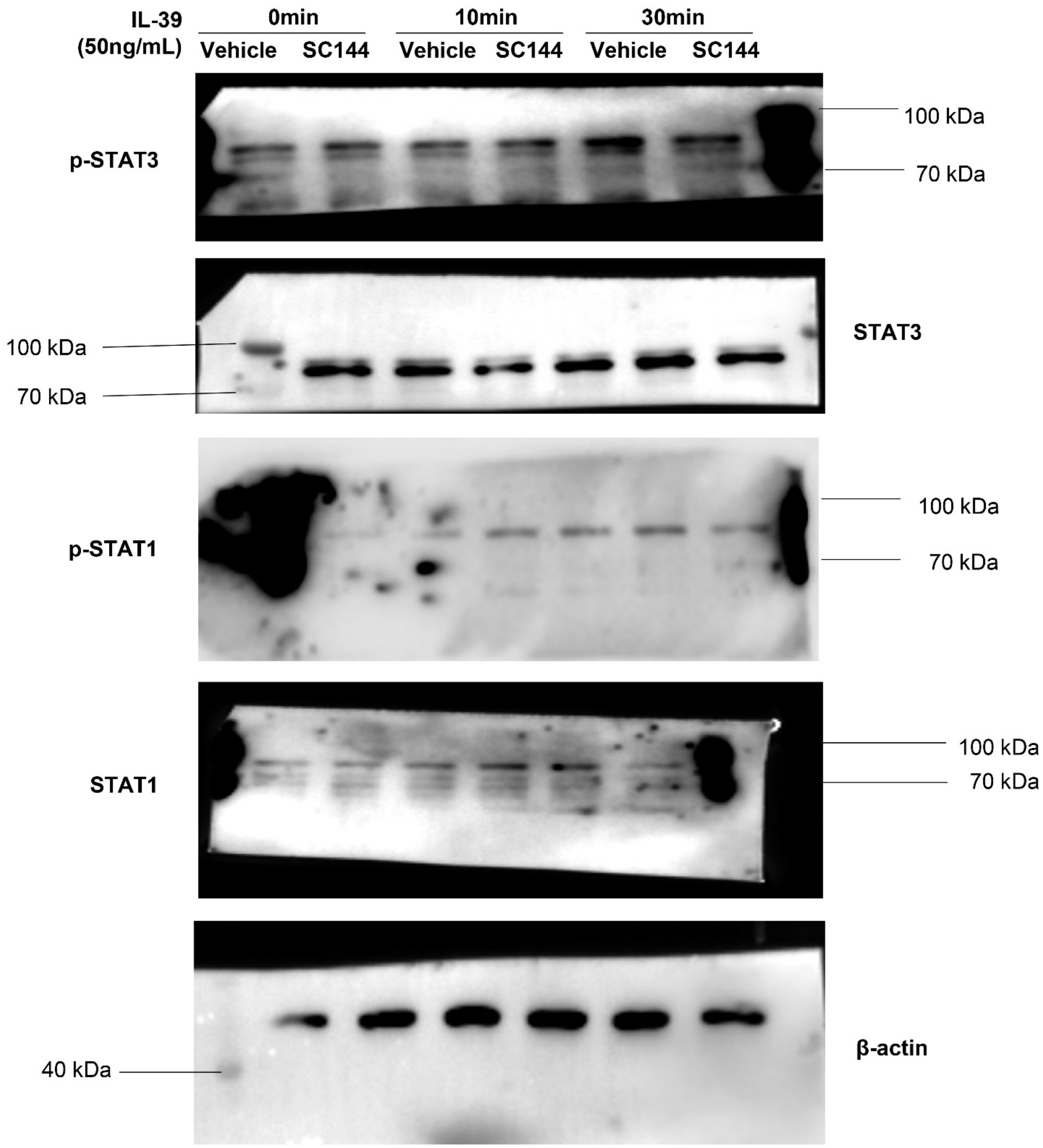

Figure S10. Unprocessed, full-length blots corresponding to the western blot panels (p-STAT3, STAT3, p-STAT1, STAT1, and β-actin). Raw images for Figure 7C.

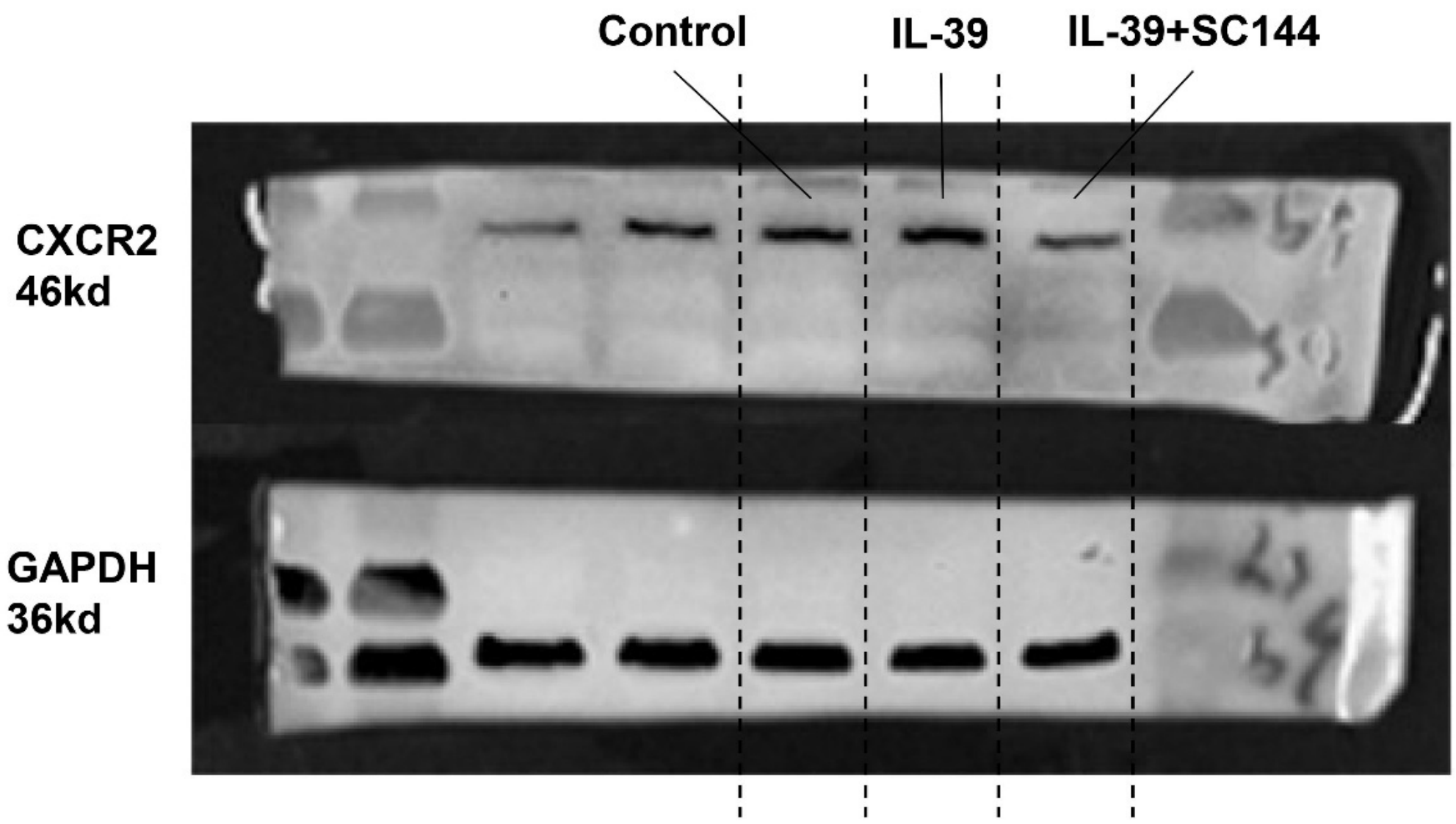

Figure S11. Unprocessed, full-length blots corresponding to the western blot panels (CXCR2 and GAPDH). Raw images for Figure 8B.
